# Intricate genetic variation networks control the adventitious root growth angle in apple

**DOI:** 10.1186/s12864-020-07257-8

**Published:** 2020-12-01

**Authors:** Caixia Zheng, Fei Shen, Yi Wang, Ting Wu, Xuefeng Xu, Xinzhong Zhang, Zhenhai Han

**Affiliations:** College of Horticulture, China Agricultural University, Beijing, 100193 China

**Keywords:** *Malus*, RGA, BSA-seq, RNA-seq, QTLs, Genetic variation

## Abstract

**Background:**

The root growth angle (RGA) typically determines plant rooting depth, which is significant for plant anchorage and abiotic stress tolerance. Several quantitative trait loci (QTLs) for RGA have been identified in crops. However, the underlying mechanisms of the RGA remain poorly understood, especially in apple rootstocks. The objective of this study was to identify QTLs, validate genetic variation networks, and develop molecular markers for the RGA in apple rootstock.

**Results:**

Bulked segregant analysis by sequencing (BSA-seq) identified 25 QTLs for RGA using 1955 hybrids of the apple rootstock cultivars ‘Baleng Crab’ (*Malus robusta* Rehd., large RGA) and ‘M9’ (*M. pumila* Mill., small RGA). With RNA sequencing (RNA-seq) and parental resequencing, six major functional genes were identified and constituted two genetic variation networks for the RGA. Two single nucleotide polymorphisms (SNPs) of the *MdLAZY1* promoter damaged the binding sites of MdDREB2A and MdHSFB3, while one SNP of *MdDREB2A* and *MdIAA1* affected the interactions of MdDREB2A/MdHSFB3 and MdIAA1/MdLAZY1, respectively. A SNP within the *MdNPR5* promoter damaged the interaction between *MdNPR5* and MdLBD41, while one SNP of *MdLBD41* interrupted the MdLBD41/MdbHLH48 interaction that affected the binding ability of MdLBD41 on the *MdNPR5* promoter. Twenty six SNP markers were designed on candidate genes in each QTL interval, and the marker effects varied from 0.22°-26.11°.

**Conclusions:**

Six diagnostic markers, SNP592, G122, b13, Z312, S1272, and S1288, were used to identify two intricate genetic variation networks that control the RGA and may provide new insights into the accuracy of the molecular markers. The QTLs and SNP markers can potentially be used to select deep-rooted apple rootstocks.

## Background

Apple (*Malus domestica* Borkh.) is one of the predominant fruit crops. Dwarfing rootstock is widely used as an ideal method for achieving high-density planting of apple [1]. However, apple trees that are grafted on dwarfing rootstocks usually have a relatively shallow root system, which leads to poor anchorage and increased sensitivity to ambient stress from, for example, wind or water logging [2]. Moreover, plants with shallow roots are often vulnerable to environmental stresses such as drought, cold, and salinity [3, 4], whereas deep roots are pivotal for water uptake, nutrient uptake, and adaptability [5]. The depth of the root architecture is determined by the root growth angle (RGA), which is defined as the angle between the direction of root growth and the horizontal axis [6, 7]. However, the understanding of the development and underlying mechanisms of the RGA in apple lag far behind that in cereal crops [8–10].

Gravity, light, and water potential are major environmental factors that affect the RGA [11]. The perception and response of plant roots to gravity have been described as gravitropic [12, 13]. Two different mechanisms of gravity signal transduction are active in root columella cells and in the root elongation zone [14]. The starch-statolith hypothesis indicates that the columella cells of plant roots sense the direction of gravity by sedimentation of amyloplasts and can thus maintain positive gravitropism. Consequently, amyloplasts are always at the cell bottom matching the direction of gravity [15, 16]. A different gravitropic sensing hypothesis states that the cells of the root elongation zone also sense gravity and bend following the direction of gravity without the use of amyloplasts [14]. The RGA is also easily affected by light, which suggests that phototropism governs the RGA [17]. The RGA in phytochrome A (*phyA*) mutants of *Arabidopsis* responds positively to red light and is negatively phototropic to blue light in phototropin1 (*phot1*) mutants [18–20]. However, water potential is another factor that affects the RGA, and this response of plant roots is often called hydrotropism [21, 22]. Several mutants in *Arabidopsis*, such as *none hydrotropic response1* (*nhr1*) or *mizu-kussei1* (*miz1*), and *altered hydrotropic response1* (*ahr1*), demonstrated varied hydrotropic responses to water gradients [21, 23–25]. LONG HYPOCOTYL 5 (HY5)-mediated light signalling has been shown to enhance the hydrotropism of *MIZ1*, which initiates downward growth of plant roots and leads to the development of a larger root system in *Arabidopsis* [26].

The mechanism of root gravitropism has demonstrated that on the opposite sides of plant roots, different levels of auxin accumulation lead to an asymmetric elongation zone of cells, which causes gravitropic bending of the roots [13, 27, 28]. In response to this gravitropic bending of roots, the auxin-influx carrier AUXIN RESISTANT 1 (AUX1) and auxin-efflux carriers PIN2, PIN3, and PIN7 are the central players [29–33]. *Arabidopsis* mutants with defective gravitropic responses are generated via genetic mutations in the auxin signalling pathway, mediated by interactions of both AUXIN (AUX)/INDOLE-3-ACETIC ACID (IAA) and AUXIN RESPONSE FACTOR (ARF) [34–37].

Among the mutations causing defective gravitropic responses in many plant species, *lazy1* mutations often lead to an asymmetric auxin distribution in either roots or shoots [38–42]. The functions of LAZY1 can be affected via interaction with IAA1, HEAT STRESS TRANSCRIPTION FACTOR 2D (HSFA2D), and Brevis Radix 4 (BRXL4) by altering the responses to auxin signalling [43–45]. *OsIAA* mutants in rice (*Oryza sativa* L.) have a single amino acid substitution in the core sequence of domain II, which blocks auxin signalling, and the resulting mutant plants displayed phenotypes defective in gravitropic responses [46–48]. Overexpression of the dehydration-responsive element-binding protein 2A (DREB2A) gene, which specifically binds to the sequence 5′-[A/G]CCGAC-3′, led to more vertical roots in transformants than in the wild type (WT) [49, 50]. Lateral organ boundary domain-containing proteins (LBD) are dominant regulators of the formation of adventitious roots and the development of other lateral organs [51–53]. In *Arabidopsis*, LBD adaxial-abaxial polarity can be affected by *35S*:BOP1 transgenesis, and the resulting transformants exhibited a downward-orienting silique phenotype [54, 55]. The interaction of basic helix-loop-helix 48 (bHLH48) with the C-terminus of LBD may affect the capability of LBD to bind to the target gene promoter sequence 5′-(G)CGGC(G)-3′ and may thus regulate the development of lateral organ boundaries [56–59].

To identify natural genetic variations that are involved in plant RGA, many quantitative trait loci (QTLs) have been reported in a diversity of Poaceae species, such as *Zea* hybrids [9], sorghum (*Sorghum bicolor* L.) [10, 60], rice [7, 8, 61–64], and wheat (*Triticum aestivum* L.) [65, 66]. Recently, in rice, several candidate genes, such as *DRO1*, *DRO2*, and *qSOR1*, were predicted within QTL regions on chromosomes 9, 4, and 7, respectively [61–63]. However, QTLs for RGA have not been identified in woody perennials, and the molecular genetic control of RGA remains to be elucidated.

Marker-assisted selection (MAS) aims to predict the performance of a trait of an offspring by one or few markers with major effects [67]. Root morphology and drought resistance in Kalinga III rice were greatly enhanced by MAS in a backcross population [68]. Four root trait QTLs were introduced into upland rice by using MAS to increase yield [69]. In a scenario where a trait is associated with up to thousands of genes with minor or infinitesimal effects, MAS will be ineffective. Thus, the genomic selection (GS) strategy emerged, which considers the impact of all available genetic markers for the prediction of breeding value [70]. The advantage of GS is to estimate the effects of all the markers on the target trait, e.g., genomic breeding value estimation [71, 72]. Conventionally detected significant QTLs may have distinct large effects in GS models. Significant root trait-associated QTLs have been detected in *Brassica napus* [73, 74]. Five significant root length QTLs were identified across four chromosomes in wheat by using genome-wide association mapping (GWAS) with a total of 25,125 marker pairs [75]. In contrast, the large effect markers related to root architecture were also selected in wheat by using a mixed linear model of GWAS with single nucleotide polymorphism (SNP) arrays [76].

The objective of this study was to identify QTLs and subsequent candidate genes that are associated with apple rootstock RGA via BSA-seq. Then, the interactions between candidate genes were verified to obtain diagnostic markers and to decode the genetic variation network of the RGA. Furthermore, the effects of QTL-based markers on RGA were estimated.

## Results

### Inheritance of apple adventitious RGA and construction of extreme RGA phenotype bulks

Phenotype data, in the form of the average RGA of three leafy cuttings of 1955 and 1383 hybrids derived from ‘Baleng Crab (BC)’ (*Malus robusta* Rehd.) × ‘M9’ (*M. pumila* Mill.), were obtained in 2016 and 2017, respectively (Table S1). The RGA of the hybrids segregated significantly by 9.26–58.95° (Table S1). The frequency distribution was not a typical Gaussian distribution, and the broad-sense heritability was 85.26% in both years (Fig. S1A and B).

Using the total mean RGA of the two experimental years, genomic DNA of 30 hybrids with extremely large mean RGAs (53.43°) and 30 hybrids with extremely small mean RGAs (12.70°) were mixed to construct two extreme bulks. The mean RGA values of the two extreme bulks were significantly different from each other (Fig. S1C and D).

### Identification of QTLs for RGA

A total of 237,892,668 clean reads were identified in both extreme RGA bulks (Table S2). In small and large RGA bulks, 97.38 and 96.86% of the clean reads were mapped to the *Malus* × *domestica* genome GDDH13_v1.1 (GDDH13, https://iris.angers.inra.fr/gddh13/), respectively. Only uniquely mapped reads were used for further analysis at mapping rates of 75.83 and 78.46% for small and large RGA bulks, respectively (Table S2).

Twenty-five significant QTLs were identified according to the criterion of a false discovery rate (FDR) < 0.01 (Table S3). Eleven of these QTLs were mapped on the maternal parent ‘BC’ (headed by B), eight were mapped on the pollen parent ‘M9’ (headed by M), and six were located on both parents (headed by H), with significant thresholds of 2.93, 3.13, and 3.38, respectively (Fig. S2; Table S3).

By changing the sliding window sizes of 1.25, 1.00, 0.75, 0.50, and 0.25 Mb (mega base pair), the stabilities of the QTL regions were verified, and the QTL intervals were narrowed from 31.34 to 28.14 Mb. The average G’ scores increased from 3.55 to 7.01 (Table S3).

### Analysis of transcriptome data

RNA sequencing (RNA-seq) yielded 19,935,274 clean reads from the 24 samples derived from cuttings of each of the three ‘BC’ × ‘M9’ hybrids with extremely small or extremely large RGAs. Samples were assessed at 0, 7, 14, and 21 d after cutting. Of these reads, more than 82.73% were uniquely mapped to the apple genome GDDH13 (Table S4, S5). In the RNA-seq data of extremely small and large RGAs, the correlation coefficients of three analogous hybrids were 0.72–0.97 at the same sampling time points (Table S6).

A total of 2849 differentially expressed unigenes (DEGs) with 2-fold changes (log 2 > 1 or log 2 < − 1 and FDRs < 0.05 between samples were found based on their fragments per kilobase per million mapped reads (FPKM) values in both extreme bulks during the four sampling time points. A total of 1239 (43.49%), 296 (10.39%), 330 (11.58%), and 1257 (44.12%) DEGs were distributed in samples from 0, 7, 14, and 21 d, respectively (Fig. S3A).

In the gene ontology (GO) classification, 1225 DEGs (43.00%) were identified, and more than half of these (54.68%) were enriched in the molecular function category (Fig. S4). Moreover, 706 (78.88%) DEGs were classified into metabolic pathways according to the Kyoto Encyclopedia of Genes and Genomes (KEGG) classification (Fig. S5A and B). In the KEGG enrichment analysis, 11 pathways were selected via *P*-values < 0.01, including four pathways that were closely associated with RGA, plant hormone signal transduction (60 DEGs), starch and sucrose metabolism (52 DEGs), terpenoid backbone biosynthesis (20 DEGs), and alpha-linolenic acid metabolism (21 DEGs) (Fig. S5C and D).

Of the 60 DEGs in the plant hormone signalling pathway, 30 were involved in auxin signalling (Table S7). Twenty-one auxin signalling genes (including *SAUR*, *AUX/IAA*, *GH3*, and *TIR1*) and two gibberellin signalling-related phytochrome interacting factor genes (MD09G1146000 and MD17G1132600) had significantly higher expression levels (log 2 > 1 and FDR < 0.05) in samples with large RGAs than in samples with small RGAs at 0 d (Fig. S3B; Table S7). Furthermore, two auxin signalling-related *ARF* and *LAX* genes (MD15G1014400 and MD12G1162400), two cytokinin signalling-related ARR-B family genes (MD13G1108300 and MD16G1159400), and four abscisic acid-related protein phosphatase 2C genes (MD01G1220800, MD03G1085400, MD07G1291000, and MD11G1093100) were significantly less expressed (log 2 < − 1 and FDR < 0.05) in samples with large RGAs than in those with small RGAs at 0 d (Fig. S3B; Table S7). However, three auxin signalling genes related to AUX/IAA and GH3 genes (MD09G1208000, MD17G1189100 and MD05G1092300), two cytokinin signalling-related histidine-containing phosphotransferase protein genes (MD03G1272900 and MD11G1293900), and three abscisic acid receptor PYR/PYL family genes (MD01G1158500, MD07G1227100, and MD12G1178800) had significantly higher expression in cuttings with large RGAs at 21 d (Fig. S3B; Table S7).

Of the 52 DEGs in the starch and sucrose metabolism pathways, 13 were significantly highly expressed at 0 d, whereas 15 were significantly less expressed in large RGA cuttings at 0 d. However, eight and five genes had significantly lower and higher expression levels in large RGA cuttings at 21 d, respectively (Fig. S3C; Table S7). Eighteen of the 20 DEGs related to terpenoid backbone biosynthesis were expressed at significantly higher levels in large RGA cuttings at 21 d (Fig. S3D; Table S7). Of the 21 DEGs involved in alpha-linolenic acid metabolism, nine and seven were significantly highly expressed in large RGA cuttings at 0 d and 21 d, respectively (Fig. S3E; Table S7).

To link the DEGs and the candidate genes from the QTL intervals, a co-expression network was analysed using AppleMDO tools (http://bioinformatics.cau.edu.cn/AppleMDO/?from=groupmessage) (Table S8). The SAUR-like auxin-responsive gene MD10G1060900, within the interval of QTL M10.1, was positively co-expressed with seven auxin-responsive DEGs at 0 d. Two genes from QTL B03.1, MD03G1292900, and MD03G1288700, which encode a WRKY family transcription factor and a photolyase/blue-light receptor 2, were positively co-expressed with two gibberellin-responsive and two terpenoid backbone biosynthesis-related DEGs at 0 d. A CBL-interacting protein kinase 21 gene, MD13G1265500, which is located at the QTL region of H13.1, may positively regulate four DEGs in starch and sucrose metabolism.

### Prediction of candidate genes

From the narrowed regions of the 25 identified QTLs, 2645 genes with 87,576 SNPs and 250 structural variations (SVs) were selected from parental resequencing data (Table S9). Of these genes, 576 were excluded because their SNPs or SVs did not affect the cis-element on the upstream sequence or the functional domain on the coding region (Table S9). Another 536 genes were not expressed throughout the stem tissue, and 421 genes, with SNPs or SVs only within the promoter, did not show differential expression between cuttings with large and small RGAs (Table S9). Additionally, 828 genes were excluded from further analysis since their functional annotation or subcellular localization were not closely related to the RGA, as identified via the UniProt database (http://www.uniprot.org/) (Table S9). Finally, 284 genes were selected as candidates that are likely involved in RGA (Table S10). Based on the functional annotation and the published data from the other authors [41, 43, 44, 49, 51, 55], the following six genes are potentially involved in the regulation of the RGA and thus were selected for further functional analysis: *MdNPR5* (MD09G1083600), *MdLBD41* (MD09G1088700), *MdLAZY1* (MD13G1122400), *MdbHLH48* (MD14G1064200), *MdDREB2A* (MD17G1089700), and *MdIAA1* (MD17G1198300).

### Experimental validation of candidate genes

#### Allelic variation of *MdLAZY1* positively affects the RGA

**Fig. 1:**
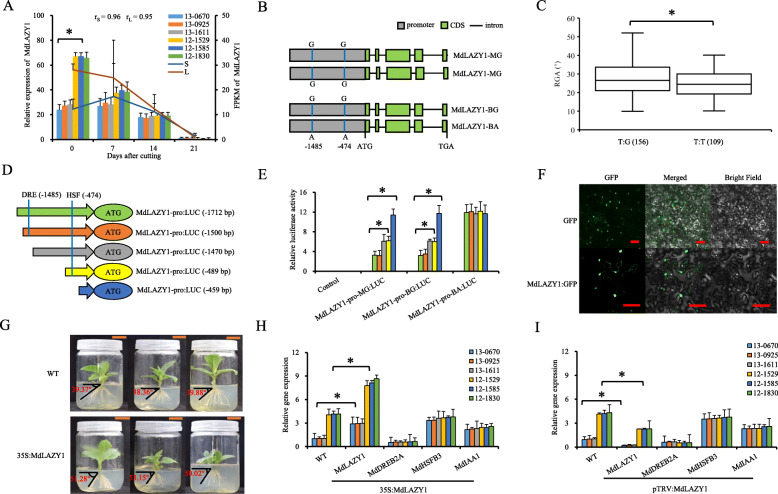
Verification of the allelic variations in the promoter of *MdLAZY1* and functional analysis of ‘Baleng Crab (BC)’ (*Malus robusta*), ‘M9’ (*M. pumila*) and their F1 hybrids. **a** *MdLAZY1* expression by real-time quantitative PCR (RT-qPCR) (bar chart) and mean fragments per kilobase per million mapped reads (FPKM) values (line chart) of the stem tissue of leafy cuttings during adventitious root formation in three hybrids (13–0670, 13–0925, and 13–1611) with small (S) and three hybrids (12–1529, 12–1585, and 12–1830) with large (L) RGAs. **b** Structural variations of *MdLAZY1*. **c** Box plot showing differences in RGAs between hybrids with the T:G and T:T genotypes of marker b13. Numbers of the hybrids are presented in parentheses. **d** Schematic representation of MdLAZY1-pro: *LUC* vectors truncated with or without DRE and HSF cis-elements. “pro” represents the promoter. **e** Transient expression of MdLAZY1-pro: *LUC* variants and truncates, which were truncated as described in panel **d**. **e** Subcellular localization of transiently expressed MdLAZY1:GFP in *Nicotiana benthamiana*. Scale bars = 50 mm. **g** RGA phenotypes in the *35S*:MdLAZY1 transgenic *N. benthamiana* lines and untransformed wild type. **h** and (**i**) Relative expression of *MdIAA1*, *MdDREB2A*, and *MdHSFB3* when *MdLAZY1* was transiently transformed by the vectors *35S*:MdLAZY1 (H) and pTRV:MdLAZY1 (**i**). Asterisks represent *P* < 0.05 by Dunnett’s multiple comparison

*MdLAZY1* was predicted near the peak of QTL B13.2 (Table S10). The expression levels of *MdLAZY1* in the stem tissue of hybrids with large RGAs were significantly higher than those in hybrids with small RGAs at 0 d after cutting (Fig. 1a). Apart from three synonymous SNPs at the coding sequence (CDS), there were also two completely linked G/A SNPs at − 1485 bp and − 474 bp upstream of the ATG codon (SNP-1485/− 474 G/A) (Fig. 1b; Table S11; Supplementary File 1). These two SNPs alter the cis-elements of DRE and HSF within the promoter of *MdLAZY1*. Kompetitive allele-specific PCR (KASP) assay of 265 F1 hybrids showed that the RGA of hybrids with the T:G genotype of marker b13, which was linked with SNP-1485/− 474 G:A, was significantly higher than those with the T:T genotype of b13, which was linked with the SNP-1485/− 474 G:G (Fig. 1c; Table S12).

Truncating the DRE cis-element or both DRE and HSF cis-elements led to an additive increase in the luciferase (LUC) activities of MdLAZY1-pro-MG:*LUC* and MdLAZY1-pro-BG:*LUC*, rather than MdLAZY1-pro-BA:*LUC* (Fig. 1d and e). These data indicated that the cis-element of DRE and HSF negatively affected the expression of *MdLAZY1.* Allele A of SNP-1485/− 474 caused functional deficiency in DRE and HSF cis-elements and increased the expression levels of *MdLAZY1* and the RGA.

Subcellular localization showed that MdLAZY1 was localized in the nucleus and plasma membrane (Fig. 1f). Moreover, *35S*:MdLAZY1 transgenic *Nicotiana benthamiana* lines were obtained and confirmed by PCR at the DNA and cDNA levels (Fig. S6A). A significant increase in RGA (by a mean of 12.28°) was observed in *35S*:MdLAZY1 transformants compared with that of the WT (Fig. 1g). When *35S*:MdLAZY1 and *pTRV*:MdLAZY1 were transiently transformed into hybrids from ‘BC’ × ‘M9’ with small and large RGAs, the relative expression levels of *MdMdIAA1*, *MdDREB2A*, and *MdHSFB3* did not show significant changes compared with those of untransformed hybrids (Fig. 1h and i). These data indicated that *MdLAZY1* functions downstream of *MdMdIAA1*, *MdDREB2A*, and *MdHSFB3*.

#### Allelic variations of *MdIAA1*, *MdDREB2A*, and *MdHSFB3*

**Fig. 2:**
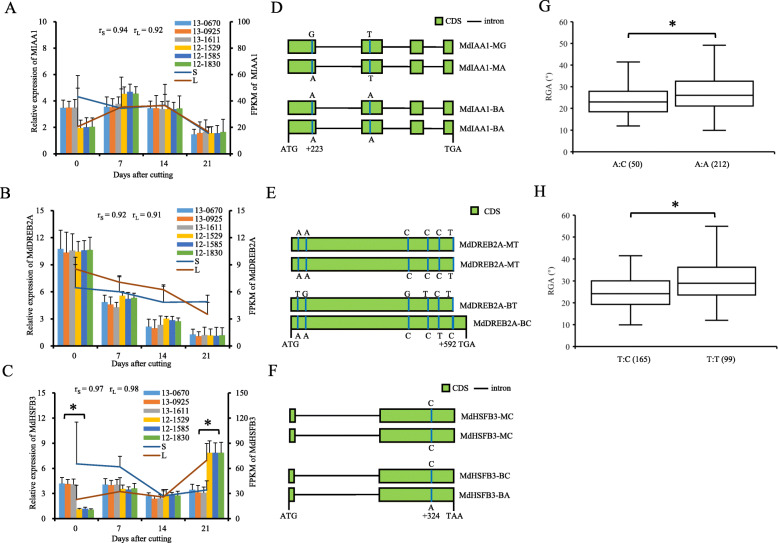
Allelic variations and the expression patterns of *MdIAA1*, *MdDREB2A*, and *MdHSFB3* in ‘BC’, ‘M9’ and their F1 hybrids. **a**-**c** Expression of *MdIAA1* (**a**), *MdDREB2A* (**b**), and *MdHSFB3* (**c**) by RT-qPCR (bar chart) and mean FPKM values (line chart) during adventitious root formation in hybrids with small (S) (13–0670, 13–0925, and 13–1611) and large (L) (12–1529, 12–1585, and 12–1830) RGAs, respectively. (D-F) Schematic of single nucleotide variations of *MdIAA1* (**d**), *MdDREB2A* (**e**), and *MdHSFB3* (**f**). (**g**) and (**h**) Box plots showing RGA differences of markers G122 (**g**) and SNP592 (**h**), respectively. Numbers of the hybrids are given in parentheses. Asterisks represent P < 0.05 by Dunnett’s multiple comparison

MdIAA1 (QTL M17.1) showed significant protein sequence alignment with IAA17 of *Arabidopsis thaliana* (https://www.arabidopsis.org/Blast/index.jsp) (e-value = 4e-63) (Table S10). No significant differences in the relative expression of *MdIAA1* were detected 0–21 d after cutting between stem tissues of F1 hybrids with large and small RGAs (Fig. 2a). The full-length CDS of *MdIAA1* comprises four exons, encoding a 205-aa protein. An A/G variant was detected at + 223 bp downstream of the ATG codon (SNP223 A/G). SNP223 resulted in an amino acid substitution from lysine to glutamate at the conserved domains. Another SNP274 A/T at the second exon (which causes amino acid substitution) was homozygous A:A in ‘BC’ and homozygous T:T in ‘M9’. Consequently, SNP274 may not segregate in the F1 population (Fig. 2d; Table S11; Supplementary File 2A and B). The RGA of 50 hybrids with the A:C genotype of the KASP marker G122, which was linked with SNP223 A:G, was significantly lower than that of 212 hybrids with the A:A genotype of G122, which was linked with SNP223 A:A (Fig. 2g; Table S12).

The *MdDREB2A* gene was predicted from QTL B17.1 (Table S10). No significant differences were detected in the relative expression of *MdDREB2A* between cuttings of F1 hybrids with small and large RGAs at any time point after cutting (Fig. 2b). The *MdDREB2A* gene, which did not contain an intron, had the following six nonsynonymous SNPs: SNP25 A/T, SNP27 A/G, SNP425 C/G, SNP515 C/T, SNP551 C/T, and SNP592 T/C. Of these six SNPs, SNP592 caused a stop loss variation with a five amino acid extension (Fig. 2e; Table S11; Supplementary File 2C and D). Significantly smaller RGA values were detected in 165 hybrids with T:C than in 99 hybrids with the T:T genotype of SNP592 (Fig. 2h).

*MdHSFB3* (MD14G1075400) was not located in the intervals of any QTL. The expression of *MdHSFB3* was higher at day 0 but was lower at day 21 after cutting in the stem tissues of hybrids with small RGAs than in those with large RGAs (Fig. 2c). *MdHSFB3* encodes a 163-aa protein, and SNP324 C/A of *MdHSFB3* resulted in an amino acid substitution from aspartate to glutamate (Fig. 2f; Table S11; Supplementary File 2E and F).

#### Non-allelic interaction of *MdLAZY1*, *MdIAA1*, *MdDREB2A*, and *MdHSFB3*

**Fig. 3:**
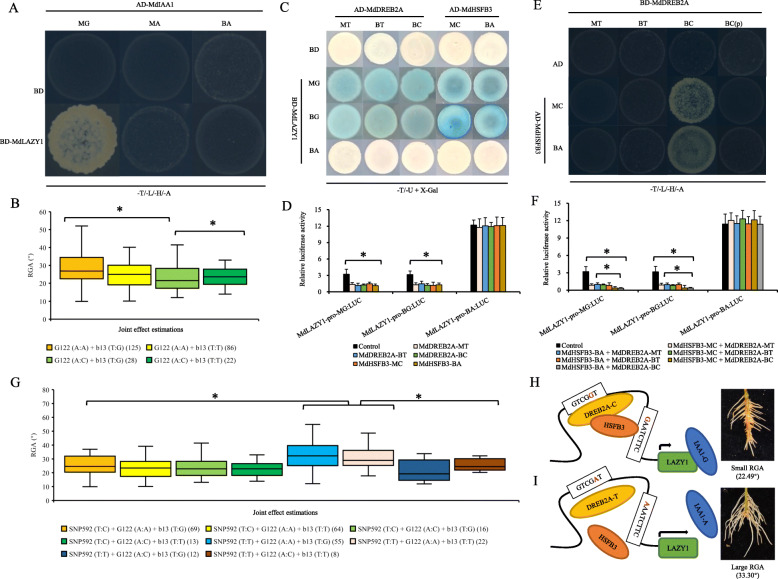
Non-allelic interactions between *MdLAZY1*, *MdIAA1*, *MdDREB2A*, and *MdHSFB3* and their effects on adventitious RGA. **a** Yeast two-hybrid (Y2H) assay showing protein-protein interactions between MdLAZY1 and MdIAA1. **b** and (**g**) Joint genotype effect estimations of markers G122/b13 (**b**) and G122/b13/SNP592 (**g**). Numbers of the hybrids are presented in parentheses. **c** Yeast-one-hybrid (Y1H) images showing the interactions of the MdLAZY1 promoter with the proteins MdDREB2A and MdHSFB3. **d** Transient coexpression analysis of MdLAZY1-pro: *LUC* interacting with *35S*:MdDREB2A or *35S*:MdHSFB3. **e** Y2H assay showing protein-protein interactions between MdDREB2A and MdHSFB3. *BD-*DREB2A-BC (p) indicates a point mutation of C (SNP592) to T in MdDREB2A-BC. **f** Transient coexpression analysis of MdLAZY1-pro: *LUC* interacting with *35S*:MdDREB2A and *35S*:MdHSFB3. Asterisks represent P < 0.05 by Dunnett’s multiple comparison. **h** and (**i**) Proposed model for the genetic variation network regulating RGA. The arrow length indicates the gene expression level. Numbers of the hybrids are presented in parentheses

A yeast two-hybrid (Y2H) assay and bimolecular fluorescence complementation (BiFC) showed that MdLAZY1 interacted with MdIAA1-MG but not with MdIAA1-MA or MdIAA1-BA (Fig. 3a; Fig. S7A). The joint effect of *MdLAZY1* SNP-1485/− 474 G:A and *MdIAA1* SNP223 A:G was − 4.85°, which is far lower than their individual effects (Fig. 3b; Table S13). These data indicated an obvious epistasis of the *MdIAA1* SNP223 G allele on allele A of SNP-1485/− 474 in the *MdLAZY1* promoter.

*BD*-MdLAZY1-MG or *BD*-MdLAZY1-BG (but not *BD*-MdLAZY1-BA) interacted with all genotypes of *AD*-MdDREB2A and *AD*-MdHSFB3 as identified by yeast-one-hybrid (Y1H) assay (Fig. 3c). Coinjection of *35S*:MdDREB2A or *35S*:MdHSFB3 with MdLAZY1-pro-MG:*LUC* and MdLAZY1-pro-BG:*LUC*, instead of MdLAZY1-pro-BA:*LUC*, resulted in lower LUC activity than that of the control (Fig. 3d). These data further validated that MdDREB2A and MdHSFB3 negatively regulated the expression of *MdLAZY1* via the cis-element of DRE and HSF.

MdDREB2A-BC, rather than MdDREB2A-BT or MdDREB2A-MT, interacted with MdHSFB3-BA and MdHSFB3-MC, as identified via Y2H and BIFC assays. However, a point mutation of MdDREB2A-BC (592 C to T), MdDREB2A-BC (p), could not interact with MdHSFB3 at all (Fig. 3e and Fig. S7B). These results indicated that the SNP592 T/C of MdDREB2A altered its ability to interact with MdHSFB3. Coinjection of MdLAZY1-pro-MG:*LUC* or MdLAZY1-pro-BG:*LUC* (but not MdLAZY1-pro-BA:*LUC*) with *35S*:MdHSFB3-BA/MC + *35S*:MdDREB2A-BC led to significantly lower LUC activity compared with both the control and samples coinjected with *35S*:MdHSFB3-BA/MC + *35S*:MdDREB2A-BT/MT (Fig. 3f). The interaction between MdDREB2A SNP592 allele C and MdHSFB3 enhanced the negative regulation of *MdLAZY1* promoter activity of SNP-1485/− 474 allele G.

The combination of the SNP223 A:A of *MdIAA1* and the SNP592 T:T of *MdDREB2A* exhibited larger genotype effects (4.82° and 6.14°) than those with one or two heterozygous genotypes, irrespective of *MdLAZY1* genotype (Fig. 3g, Table S13). Therefore, *MdDREB2A* and *MdIAA1* exhibited epistatic non-allelic effects on *MdLAZY1* (Fig. 3h and i).

#### Allelic variation of *MdNPR5* negatively affects RGA

**Fig. 4:**
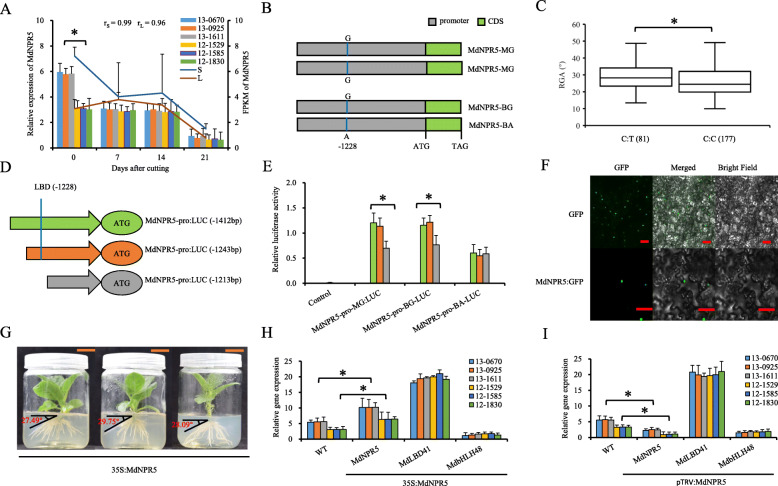
Allelic variation of *MdNPR5*, effects on promoter activity, and function in adventitious RGA in ‘BC’, ‘M9’ and their F1 hybrids. **a** *MdNPR5* expression by RT-qPCR (bar chart) and mean FPKM values (line chart) during adventitious root formation in hybrids with small (S) (13–0670, 13–0925, and 13–1611) and large (L) (12–1529, 12–1585, and 12–1830) RGAs. **b** Schematic representation of single nucleotide variation within the promoter of *MdNPR5*. **c** Box plots showing differences in RGA between hybrids with the C:T and C:C genotypes of marker S1272. Numbers of hybrids are presented in parentheses. **d** Schematic of promoter truncations of MdLNPR5-pro: *LUC* vector constructs with or without the LBD cis-element. **e** Transient expression analysis of various MdNPR5-pro: *LUC* constructs. “pro” represents the promoter. **f** Subcellular localization of transiently expressed MdNPR5:GFP in *N. benthamiana* leaves. Scale bars = 50 mm. **g** RGA phenotypes of the *35S*:MdNPR5 transgenic *N. benthamiana* lines and untransformed wild type. The RGA values are presented in parentheses. **h** and (**i**) Relative expression of *MdLBD41* and *MdbHLH48* when *MdNPR5* was transiently expressed by the vectors *35S*:MdNPR5 (**h**) and pTRV:MdNPR5 (**i**). Asterisks represent P < 0.05 by Dunnett’s multiple comparison

*MdNPR5* was in QTL B09.1 (Table S10). The relative expression of *MdNPR5* was significantly higher in the stem tissues of cuttings with small RGAs than in those with large RGAs at day 0 after cutting (Fig. 4a). *MdNPR5* encoded a 61-aa protein without nonsynonymous variations in the unique exon. However, SNP-1228 G/A, which alters the LBD cis-element, was found within the promoter of *MdNPR5* (Fig. 4b; Table S11; Supplementary File 3). The KASP assay showed that the average RGA of 81 F1 hybrids with the SNP-1228 G:A genotype, linked by the C:T of marker S1272, was significantly larger than that of 177 hybrids with the SNP-1228 G:G genotype, linked with the C:C of marker S1272 (Fig. 4c; Table S12).

A promoter truncation assay showed that the LUC activity of both full-length and truncated MdNPR5-pro-MG and MdNPR5-pro-BG (including − 1228 G) was significantly higher than that of MdNPR5-pro-BA and truncated the − 1228 G allele in MdNPR5-pro-MG and MdNPR5-pro-BG (Fig. 4d and e). These data indicated that the LBD cis-element exerted a positive effect on *MdNPR5* transcription and that SNP-1228 allele A completely destroyed the LBD cis-element.

Subcellular localization analysis identified GFP signals of the *MdNPR5* fused protein in the nucleus (Fig. 4f). In addition, the *35S*:MdNPR5 transgenic *N. benthamiana* lines exhibited a significant decrease in RGA (10.76° by mean) compared with that of the WT (Fig. 1g; Fig. 4g; Fig. S6B). The *35S*:MdNPR5 and *pTRV*:MdNPR5 transiently transformed hybrids with small and large RGAs from ‘BC’ × ‘M9’ did not show significant differences in the relative expression levels of *MdMdLBD41* and *MdbHLH48* compared with those of untreated hybrids (Fig. 4h and i). These data indicated that *MdNPR5* acted downstream of *MdMdLBD41* and *MdbHLH48*.

#### Allelic variations and effects of *MdLBD41* and *MdbHLH48* on RGA

**Fig. 5:**
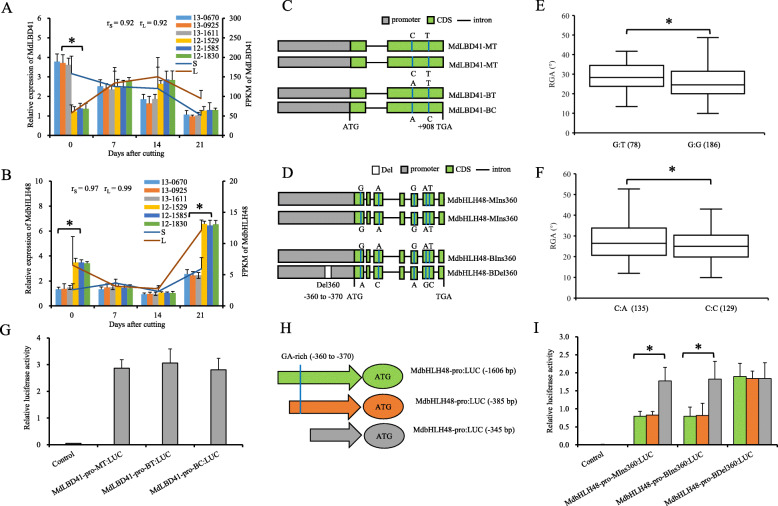
Allelic variation, expression of *MdLBD41* and *MdbHLH48*, and their effects on adventitious RGA in ‘BC’, ‘M9’, and their F1 hybrids. **a** and (**b**) Expression of *MdLBD41* (**a**) and *MdbHLH48* (**b**) by RT-qPCR (bar chart) and mean FPKM values (line chart) in the stem tissue during adventitious root formation in leafy cuttings of three hybrids with small (S) (13–0670, 13–0925, and 13–1611) and large (L) (12–1529, 12–1585, and 12–1830) RGAs. (C) and (D) Schematic diagram of variations of *MdLBD41* (**c**) and *MdbHLH48* (**d**). **e** and (**f**) Box plots showing RGA differences of markers Z312 (**e**) and S1288 (**f**). The numbers of the hybrids are presented in parentheses. **g** Transient expression analysis of various constructs of MdLBD41-pro: *LUC*. “pro” represents the promoter. **h** Schematic of MdbHLH48-pro: *LUC* vector constructs truncated either with or without Del360. **i** Transient expression analysis of various truncated constructs of MdbHLH48-pro: *LUC*. Asterisks represent significance levels of P < 0.05 by Dunnett’s multiple comparison

*MdLBD41* was located at the peak of QTL B09.1 (Table S10). *MdLBD41* had significantly higher relative expression and FPKM values in the stem tissue of hybrids with small RGAs than in those with large RGAs at 0 d after cutting (Fig. 5a). The full-length CDS of *MdLBD41* comprises two exons, which encode a 305-aa protein. SNP908 T/C caused an amino acid substitution. Another synonymous SNP713 A/C was homozygous in both parents and did not segregate in their F1 population (Fig. 5c; Table S11; Supplementary File 4A and B). The RGAs of 78 hybrids with the G:T genotype of marker Z312, which was linked with SNP908 T:C, were significantly larger than those of 186 hybrids with the Z312 G:G genotype, which was linked with SNP908 T:T (Fig. 5e; Table S12). The promoter activity of the 1655 bp DNA sequences upstream in *MdLBD41* was similar between ‘BC’ and ‘M9’ (Fig. 5g; Supplementary File 4C). These data indicated that a SNP908 variant (rather than variations of the promoter) affected MdLBD41 function.

The relative expression and FPKM values of *MdbHLH48* were significantly higher at d 0 and 21 after cutting in the stem tissues of hybrids with large RGAs than those with small RGAs (Fig. 5b). MdbHLH48 encoded a 357-aa protein and had five closely linked non-synonymous SNPs in the CDS between ‘BC’ and ‘M9’: SNP344 G/A, SNP644 A/C, SNP785 G/A, SNP991 A/G, and SNP1006 T/C. There was also an 11-bp deletion between − 360 and − 370 bp upstream of the ATG codon (Del360), which altered the GA-rich motif in ‘BC’ but not in ‘M9’ (Fig. 5d; Table S11; Supplementary File 5). The RGA of 135 hybrids with the S1288 C:A genotype, which was linked with Ins360:Del360, was significantly larger than that of 129 hybrids with the S1288 C:C genotype, which was linked with Ins360:Ins360, according to the KASP assay (Fig. 5f; Table S12). The *MdbHLH48* promoter truncation assay showed that truncating the Ins360 allele in MIns360 and BIns360 led to a significant increase in LUC activity (Fig. 5h and i). These data indicated that GA-rich elements had a negative effect on *MdbHLH48* transcription and that Del360 completely destroyed the GA-rich element.

#### Non-allelic interactions of *MdNPR5*, *MdLBD41* and *MdbHLH48*

**Fig. 6:**
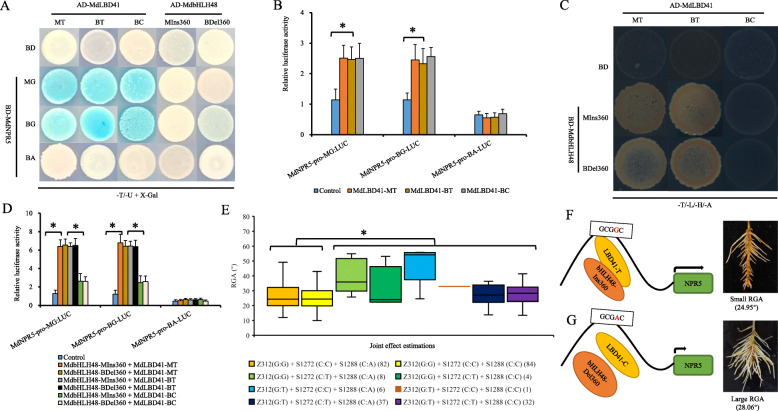
Non-allelic interactions and joint effects of *MdNPR5*, *MdLBD41*, and *MdbHLH48* natural variants on adventitious RGA in leafy cuttings of hybrids derived from ‘BC’ × ‘M9’ apple rootstock. **a** Y1H image showing the interaction of the *MdNPR5* promoter with MdLBD41 and MdbHLH48. **b** Transient coexpression assay of MdNPR5-pro: *LUC* variants interacting with *35S*:MdLBD41 variants. **c** Y2H images showing interactions between variants of MdLBD41 and MdbHLH48. **d** Transient coexpression assay of MdNPR5-pro: *LUC* interacting with *35S*:MdLBD41 and *35S*:MdbHLH48 variants. **e** Joint genotype effect estimates of markers S1272 (C/T), Z312 (T/G), and S1288 (A/C). Numbers of the hybrids are presented in parentheses. Asterisks represent P < 0.05 by Dunnett’s multiple comparison. **f** and (**g**) Proposed model for the genetic variation networks regulating apple rootstock RGA by *MdNPR5*, *MdLBD41*, and *MdbHLH48*. The arrow length indicates the gene expression level. Numbers of the hybrids are presented in parentheses

The Y1H assay showed that the protein variants MdLBD41-MT, MdLBD41-BT, and MdLBD41-BC interacted with the promoters of both MdNPR5-MG and MdNPR5-BG but not with MdNPR5-BA (Fig. 6a). Higher levels of LUC activity were detected in MdNPR5-pro-MG:*LUC* and in MdNPR5-pro-BG:*LUC* than in MdNPR5-pro-BA:*LUC* coinjected with any of *35S*:MdLBD41 variants or in the non-coinjected control (Fig. 6b). These results indicated that *MdLBD41* positively regulated the expression of *MdNPR5* via the LBD cis-element.

MdbHLH48-MIns360 and MdbHLH48-BDel360 did not interact with *MdNPR5,* according to Y1H assay (Fig. 6a). However, the Y2H and BIFC assays showed that MdLBD41-MT and MdLBD41-BT (rather than MdLBD41-BC) interacted with both MdbHLH48-MIns360 and MdbHLH48-BDel360 (Fig. 6c; Fig. S7C). This indicated that SNP908 T/C allelic variation of MdLBD41 in the C-terminus damaged the binding ability with MdbHLH48. The LUC activity was significantly lower when MdNPR5-pro-MG:*LUC* or MdNPR5-pro-BG:*LUC* (but not MdNPR5-pro-BA:*LUC*) were coinjected with MdbHLH48-BDel360 or MdbHLH48-MIns360 and MdLBD41-BC than with MdLBD41-BT or MdLBD41-MT. (Fig. 6d). These data further indicated that the lack of interaction between MdLBD41 SNP908 allele C and MdbHLH48 variants negatively affects the activity of the *MdNPR5* promoter.

*MdNPR5* and *MdLBD41* were located within the same interval of QTL B09.1; thus, both loci showed a close linkage, and 19 of 256 hybrids were recombinants (Table S14). The joint genotype effects showed that the RGAs of hybrids with the genotypes *MdNPR5* SNP-1228 G:G and *MdLBD41* SNP908 T:T were smaller (− 2.21° and − 1.18°) than those of hybrids with one or two heterozygous genotypes, irrespective of *MdbHLH48* genotypes. The *MdbHLH48* Ins360:Del360 genotype exerted a greater effect on RGA than Ins360:Ins360 (Fig. 6e, Table S14). *MdLBD41* and *MdbHLH48* exhibited epistatic non-allelic effects on *MdNPR5* (Fig. 6f and g).

### Marker genotype effect estimation

To estimate the QTL-based marker effect on RGA, 26 SNPs were selected for the design of KASP markers. These SNPs were selected on candidate genes near the peak of the abovementioned 25 QTLs (Table S15). These included six diagnostic markers, SNP592, G122, b13, Z312, S1272, and S1288, the latter five of which are tightly linked to *MdIAA1* SNP223, *MdLAZY1* SNP-1485/− 474, *MdLBD41* SNP908, *MdNPR5* SNP-1228, and *MdbHLH48* Del360 (Table S12). These markers were genotyped by KASP assay in 266 randomly chosen F1 hybrids from the ‘BC’ × ‘M9’ population (Table S16). Both the marker effects and the marker genotype effects varied considerably (Table S17). The marker Z1796 from QTL H04.1 exhibited the largest marker effect (26.11°) on RGA, whereas the marker effect (0.22°) of Z4196 from QTL M16.1 was the smallest (Table S17). The largest genotype effect was estimated for Z1796 A:A (23.53°), while the smallest genotype effect was detected for G122 C:A (− 4.00°) (Table S17). Of these six diagnostic markers, SNP592 T:T and G122 A:A displayed the largest non-allelic epistatic effects (6.14°) on b13 T:G (Table S13). The smallest non-allelic effects (− 2.21°) on S1272 C:C were clearly detected for markers Z312 G:G and S1288 C:C (Table S14).

## Discussion

### Adventitious RGA is controlled by multiple genes with varied effects in apple

The nodal root angle of sorghum measured on the 141 F6 recombinant inbred lines ranged from 14.5° to 32.3°, and the heritability was 73.7% [60]. The seminal RGA of wheat ranged from 25° to 60° in 103 double haploid lines, and the estimated heritability was 0.43 [65]. In this study, the RGA of a population of 1955 hybrids from ‘BC’ × ‘M9’ segregated continuously with an overall deviation of 9.26–58.95° and broad-sense heritability of 85.26%, which were consistent with those in sorghum and wheat and were similar to our previous findings [77]. Four to five QTLs for RGA were detected in maize, rice, and sorghum [7, 9, 60, 63, 64]. In the current study, as many as 25 QTLs of RGA were identified in apple rootstock. Of the 25 QTLs for RGA in this study, 11, 8, and 6 were of the B, M, and H type, respectively, markers of which were heterozygous for the maternal parent, the pollen parent, and both parents, respectively. These data indicated that apple RGA is relatively less affected by the environment but has more complicated multi-genetic control than that observed in cereal crops.

Adventitious root formation from stem cuttings is usually divided into three stages based on physiological and metabolic markers and can be categorized as root induction, root initiation, and root extension [1]. The first critical stage of adventitious rooting regulation occurs during adventitious root induction [78, 79]. Data in this study showed that the earliest stage at which the RGA could be determined was before day 7 since up to 40% of DEGs were identified from transcriptomic data at day 0. Moreover, most DEGs for auxin, cytokinin, gibberellin, and abscisic acid were detected at 0 d, which suggests their important roles in the regulation of the RGA. Another root developmental process that determines RGA occurs during root elongation [80]. The adventitious roots in apple rootstock undergo elongation 14–21 d after cutting [81]. In this study, 44.12% of DEGs were detected at day 21 after cutting, indicating that RGA may also be associated with root elongation.

### Natural variations involving auxin signalling and gravitropism contributed to genetic diversity in the RGA

During root formation and development processes, auxin regulates the gravitropic set-point angles by adjusting the magnitude of the anti-gravitropic offset component via TIR1/AFB-Aux/IAA-ARF-dependent auxin signalling within the gravity-sensing cells of the roots [82]. Some functional genes, *DRO1*, *LAZY1* and *qSOR1*, which regulate root gravitropism by auxin signalling, are closely associated with RGA [7, 41, 83]. In this study, 153 DEGs involved in gravitropism, auxin signalling, and related pathways were detected by RNA-seq. Similarly, from QTL intervals, candidate RGA genes (*MdLAZY1*, *MdIAA1*, *MdDREB2A*, *MdNPR5*, *MdLBD41* and *MdbHLH48*) were also involved in auxin signalling and root gravitropism.

Natural variation of the RGA has been reported, but the genetic basis for this variation is largely unknown [9, 10, 61, 66]. Introgression lines in rice demonstrated that combinations of allelic variations in *qSOR1* and *DRO1* controlled the RGA in rice [83]. To date, little is known about genetic variation in apple RGAs. The results of the present study revealed that six functional genes developed two epistatic non-allelic variation networks regulating the RGA. One epistatic non-allelic network showed that SNP-1485/− 474 of the *MdLAZY1* promoter significantly affected the expression level and RGA by altering DRE and HSF cis-elements, which interacted with MdDREB2A and MdHSFB3, respectively. Then, the SNP592-induced stop loss variation on *MdDREB2A* affected the interaction with MdHSFB3, which in turn significantly influenced *MdLAZY1* expression. The structural variation of the MdDREB2A protein affects its function, not its expression, which is consistent with a report on *Robinia pseudoacacia* [50]. IAA17 interacted with LAZY1, which affected the auxin response and gravitropism in maize [43]. A SNP in the IAA core sequence interrupted root gravitropism, and it remains unclear whether this affected the interaction with LAZY1 [43, 46–48]. In this study, SNP223 at the *MdIAA1* CDS affected the interaction with MdLAZY1 and subsequently the RGA of cuttings.

Another epistatic non-allelic network controlling RGA was formed by SNP-1228 of *MdNPR5*, SNP908 of *MdLBD41*, and Del360 of *MdbHLH48*. SNP-1228 damaged the LBD cis-element of the *MdNPR5* promoter, which affected the interaction with the MdLBD41 protein, as well as the expression of *MdNPR5* and the RGA. SNP908 in the C-terminus of *MdLBD41* also influenced the transcription activity of the target gene *MdNPR5* by altering the protein-to-protein interaction between MdLBD41 and MdbHLH48 that has been reported for *Arabidopsis* [56]. The GA-rich cis-element exerts a negative regulatory effect on its own promoter [84]. Del360 of *MdbHLH48* increased both the gene expression of *MdbHLH48* and the RGA by affecting the GA-rich cis-element. Moreover, higher *MdbHLH48* expression levels at d 0 and 21 after cutting were not consistent with the lower levels of *MdNPR5* expression, indicating the possible existence of an undiscovered regulatory mechanism.

### QTL-based markers will be a potentially efficient tool for the breeding of deep root architecture

To date, many QTL mapping studies for diverse species have provided an abundance of DNA marker-trait associations [85, 86]. To increase the selection efficiency and the predictability of genetic values, candidate genes are often predicted within the significant intervals of QTLs. The ideal marker can be designed to the functional variation loci of the candidate genes, which effectively eliminates the linkage disequilibrium decay of these QTL-based markers [87]. These markers are sometimes called diagnostic markers [88]. In this study, 26 QTL-based markers were developed, the marker effects varied widely from 0.22° to 26.11°, and the marker genotype effect varied from − 4.00 ° to 23.53 °. Diagnostic markers SNP592, G122, b13, Z312, S1272, and S1288 were closely linked with functional variation loci by experimental validation.

The predictability of QTL-based markers is likely not transferable to different populations [89, 90]. However, the markers for RGA in this study can potentially be used for selecting materials that are genetically related to ‘M9’ and ‘BC’, because both ‘M9’ and ‘BC’ have been frequently used as parental cultivars in apple rootstock breeding programmes [91–94]. Apple dwarfing rootstocks usually have a smaller RGA and relatively shallow root architecture [2]. The QTLs for dwarfing ability, DW1, DW2, and DW3, have been successfully mapped in ‘M9’ apple rootstock [92–94]. None of the 25 QTLs for RGA in this study overlapped with the DW1/DW2/DW3 QTL regions, suggesting that RGA is related but not genetically linked to dwarfing ability in apple rootstocks. These data indicated that it will be practical to select apple rootstocks with both large RGAs for better anchorage and dwarfing ability for high-density planting.

## Conclusions

A total of 25 significant QTLs were identified, 11 of which were mapped on the maternal parent ‘BC’, eight were mapped on the pollen parent ‘M9’, and six were located on both parents. Transcriptomic data showed that the RGA was determined before 7 d and after 14 d, which implied that the critical stage of RGA regulation occurs during initiation and elongation of adventitious roots. Six markers were linked to the exact loci of the functional variations of genes and were involved in two molecular regulatory pathways of adventitious RGA in apple. SNP223 of *MdIAA1*, SNP592 of *MdDREB2A*, and SNP-1485/− 474 of *MdLAZY1* formed an epistatic non-allelic network. Furthermore, Del360 of *MdbHLH48*, SNP 1228 of *MdNPR5*, and SNP908 of *MdLBD41* formed another epistatic non-allelic network. A total of 26 QTL-based markers were developed with varied marker effects and genotype effects on RGA. These markers can potentially assist future breeding programmes aimed at optimizing the RGA in apple rootstocks.

## Methods

### Plant materials and phenotyping

All plant materials were obtained from China Agricultural University. The experimental research on plants, including field investigation and sample collection, was performed under institutional guidelines in accordance with local legislation. No formal identification was performed, and no voucher was deposited in a publicly available herbarium. During May 2016 and 2017, more than 30 semi-lignified leafy cuttings (10 cm in length) were collected from each of the 1955 and 1383 F1 hybrids derived from ‘M9’ (small RGA) × ‘BC’ (large RGA), respectively. The basal end of the stem cuttings (1 cm) was immediately submerged in indole-3-butyric acid (IBA, 3000 μg/L) for 1 min and plugged in trays filled with sand in a greenhouse. The relative air humidity was controlled at 95% using a fog irrigator. Thirty d after cutting, cuttings with roots were collected, washed with tap water, and photographed. The RGAs were measured using an ImageJ 1.50 scanner (National Institutes of Health, Bethesda, MD, USA) [77]. Statistical analysis was performed using SPSS 17 software (Armonk, NY, USA).

### BSA-seq

To obtain the two extreme bulks for BSA-seq, 30 hybrids with extremely large RGAs and 30 hybrids with extremely small RGAs were selected based on two-year phenotyping data. The genomic DNA of the selected hybrids was extracted from young leaves and pooled from equal quantities (i.e., 500 ng each). The two bulked DNA samples were fragmented via sonication to 300–650 bp for library construction and were paired-end sequenced using the Illumina HiSeq X-Ten platform (Illumina, San Diego, CA, USA). Then, Burrows-Wheeler Alignment (BWA) software was used to map the clean sequencing reads to the apple genome GDDH13 [95]. The default values were used for read mapping, and only uniquely mapped reads with a minimum phred score of 20 were retained [96]. SAMtool software was used to identify SNPs and indels between the aligned sequencing reads and the reference genome [97]. The G’ value method was used to statistically analyse the allelic variations in the two extreme bulks [98]. QTLs were identified using BSA Tools for Outbreeding Species (BSATOS) software, and the QTL regions were further narrowed by using varying sliding windows [96]. Analysis of allelic variations in QTLs was performed three times (pollen, maternal, or both parents) using two parental resequencing data sets [96].

### RNA-seq analysis

Three hybrids, as three biological replicates, were randomly chosen from each of the two extreme bulks and were subjected to RNA-seq analysis. At least five cuttings of each hybrid were sampled at 0 d, 7 d, 14 d, and 21 d after cutting based on previously reported anatomical changes during the formation of adventitious roots [1, 81]. Approximately 0.5 cm of basal stem tissue was sampled, including the adventitious root zone. Total RNA was extracted by using the CTAB method [99]. The mRNA of two RNA-seq libraries was isolated from 5 μg of total RNA from each sample. Then, the NEBNext Poly (A) mRNA Magnetic Isolation Module and NEBNext Ultra Directional RNA Library Prep Kit for Illumina (New England Biolabs, Ipswich, MA, USA) were applied for the preparation of two RNA-seq libraries. The cDNA libraries were sequenced (paired-end 150) from the 5′ to the 3′ ends on the Illumina HiSeq X Ten platform (Illumina). Then, clean RNA-seq reads were mapped to the apple genome GDDH13 by HISAT2 software [100]. Transcript assembly and quantification were conducted by StringTie [101]. The DEGs between small and large RGAs were analysed by DESeq2 software [102]. GO classification and GO enrichment were analysed by Blast2GO [103]. The KEGG metabolism annotation and KEGG enrichment were analysed by KOBAS2.0 [104]. The DEG analysis of major KEGG pathways (*P* < 0.01) was assessed by online network analysis in AppleMDO [105].

### Candidate gene prediction from QTLs

The genes from each QTL interval were downloaded from the apple genome GDDH13 (https://www.rosaceae.org/) [95]. By comparing parental resequencing data, genes with identified genetic variations on the parental cultivar on which the QTL was not mapped were removed from the list [96]. Genes that were not expressed throughout and genes with SNPs or SVs only within the promoter that did not show differential expression by RNA-seq were removed. For the remaining genes, the UniProt database (http://www.UniProtuniprot.org/) was used to assess the function of the corresponding proteins. Genes that were clearly not related to the target trait were excluded. The pipeline for candidate prediction has been published previously [96, 106].

### Validation and analysis of allelic variations

The 2-kb promoter sequences and the full-length coding sequence were amplified and Sanger sequenced to validate predicted variations. The primers are listed in supplementary Table S18. Analyses of amino acid sequences were performed by DNAMAN8 software. Analyses of cis-elements were performed by the online tools Plant CARE and JASPAR [107].

### Gene expression analysis

Total RNA was extracted from stem tissues of cuttings, and cDNA was synthesized. The relative expression of candidate genes was measured by real-time quantitative PCR (RT-qPCR) [107]. Analysis of variance and Dunnett’s multiple comparison were conducted to identify differences in gene expression. MdEF1α-F and MdEF1α-R were selected as quantification controls, and the primers are listed in supplementary Table S18.

### Plasmid construction and genetic transformation

The truncated promoter/LUC fusion vectors (pGreenII 0800-LUC) of *MdLAZY1* and *MdNPR5* were constructed by digestion with Pst1 and EcoR1. The full-length cDNA of *MdDREB2A*, *MdHSFB3*, *MdLBD41*, and *MdbHLH48* was fused with the overexpression vector (pGreenII 62-SK) by digestion with EcoR1 and Kpn1. The vectors were introduced into *Agrobacterium tumefaciens* strain GV1301, and PCR was used to confirm their introduction. Then, the truncated promoter/LUC fusion vectors of *MdLAZY1* and *MdNPR5* were transformed into leaf epidermal cells of one-month-old *N. benthamiana* plants and an empty vector pGreenII 0800-LUC as a control. The fusion vectors of promoter/LUC and the overexpression vector were also coinjected into leaf epidermal cells of one-month *N. benthamiana* at a 1:1 ratio. Moreover, the coinjection of promoter/LUC fusion vectors and the empty overexpression vector were used as controls. The LUC activities were measured with three biological replicates [108]. All primers are listed in Table S18.

The overexpression vectors (PRI101-AN) of *MdLAZY1* and Md*NPR5* were constructed by digestion with Kpn1 and EcoR1. The pTRV2 vectors of *MdLAZY1* and *MdNPR5* were constructed by digestion with Kpn1 and Xho1. Then, *35S*:MdLAZY1, *35S*:NPR5, and empty vectors were introduced into *N. benthamiana* by *A. tumefaciens*-mediated transformation [81]. The RGA of transgenic lines and the WT were measured at 14 d after subculture using an ImageJ 1.50 scanner. The *35S*:MdLAZY1, *35S*:NPR5, pTRV2:MdLAZY1, pTRV2:MdNPR5, and empty vectors were transformed into each of the three hybrids chosen from both extreme bulks via vacuum infiltration [109]. Identifications of transformation lines by RT-qPCR and PCR were performed as described above, and the primers are listed in Table S18.

### Subcellular localization of MdLAZY1 and MdNPR5

The full-length cDNAs of *MdLAZY1* and *MdNPR5* (without stop codons) were fused with PRI101-GFP by digestion with Kpn1 and BamHI. The two constructed vectors were introduced into *A. tumefaciens* strain GV1301 and were transformed into leaf epidermal cells of one-month-old *N. benthamiana* plants and visualized using confocal microscopy [110]. The control was obtained by treating tobacco leaves with the empty vectors of PRI101-GFP. All primers are listed in Table S18.

### Y1H assay

The full-length cDNAs of different genotypes of *MdDREB2A*, *MdHSFB3*, *MdbHLH48*, and *MdLBD41* were fused with AD vectors (PJG4–5) by digestion with EcoR1 and Xho1. Furthermore, the promoters from different genotypes of *MdLAZY1* and *MdNPR5* were fused with BD vectors (Placzi) by digestion with EcoR1 and Xho1, respectively. The vectors with AD and BD were cotransformed into the Y1H yeast strain on SD medium without Trp and Ura (−T/−U) at 30 °C for 3 d. Then, the successful transformants were selected and grown on the previous SD medium with X-gal (−T/−U/X-gal) at 30 °C for 3 d. The primers are listed in Table S18.

### Y2H assay

The full-length cDNAs of different genotypes from *MdDREB2A*, *MdbHLH48*, and *MdLAZY1* were fused with BD vectors (pGBKT7) by digestion with EcoR1 and Sal1. At the same time, the full-length cDNAs of different genotypes from *MdLBD41*, *MdIAA1*, and *MdHSFB3* were fused with AD vectors (pGADT7) by digestion with EcoR1 and Xho1, respectively. The vectors with AD and BD were cotransformed into the Y2H yeast strain on SD medium without Leu and Trp (−L/−T) at 30 °C for 3 d. Then, successful transformants were selected and grown on SD medium without Leu, Trp, His, and Ade (−L/−T/−H-A) at 30 °C for 3–5 d. The primers are listed in Table S18.

### BiFC assay

*MdDREB2A*, *MdbHLH48*, and *MdLAZY1* without stop codons in the coding sequence were cloned into NE vectors (SPYNE) by digestion with XbaI and BamHI. *MdLBD41*, *MdIAA1*, and *MdHSFB3* without stop codons in the coding sequence were cloned into CE vectors (SPYCE) by digestion with XbaI and BamHI. The fusion plasmids were cotransformed into *N. benthamiana* leaves, and transformed leaf epidermal cells were observed by confocal microscopy [111].

### Marker genotype effect estimation by KASP assay

KASP primers were designed based on the 100-bp flanking sequences of the SNPs of candidate genes. Detailed protocols can be found in the KASP genotyping chemistry user guide and manual (http://www.lgcgenomics.com). The genomic DNAs of 266 randomly chosen hybrids from ‘BC’ × ‘M9’ were extracted, and all the abovementioned markers were genotyped using the KASP assay (LGC Genomics, Beverly, MA, USA). Fluorescence detection was conducted by using an Omega Fluorostar scanner (BMG PHERAstar, BMG LabTech, Ortenberg, Germany). The genotype data were output by ‘SNP VIEWER’ software (LGC). SNP-1485/− 474 of *MdLAZY1*, SNP223 of *MdIAA1*, SNP-1228 of *MdNPR5*, SNP908 of *MdLBD41*, and Del360 of *MdbHLH48* were sequenced by PCR to measure how close these are linked with b13, G122, S1272, Z312, and S1288. The primers are listed in Table S18. The marker genotype effects were calculated by subtracting the average RGA phenotype value of a subset of hybrids with the same genotype from the overall mean phenotype value of the complete population. The marker effects were estimated using the genotype value deviation of a specific marker.

### Statistical analysis

Differences between the control and experimental treatments were analysed by one-way analysis of variance (ANOVA) through Dunnett’s multiple comparison at a significance level of α = 0.05.

## Supplementary Information

**Additional file 1: Fig. S1** Segregation and segregant bulk construction of the adventitious root growth angle (RGA) in leafy cuttings of a hybrid population derived from ‘Baleng Crab (BC)’ (*Malus robusta*) × ‘M9’ (*M. pumila*) in 2016 and 2017. (A) and (B) Frequency distribution diagrams of RGA in 2016 (A) and 2017 (B). (C) Photographs of adventitious roots in 30-day-old leafy cuttings, showing small and large RGAs. Scale bar = 10 mm. (D) Box plots showing the RGA phenotype in the small (left) and large (right) segregant bulks. Numbers of hybrids are presented in parentheses following the genotypes below the x-axis. Error bars indicate the standard deviation, and asterisks represent *P* < 0.05 by Dunnett’s multiple comparison.

**Additional file 2: Fig. S2** Profiles of the significant quantitative trait loci (QTLs) for adventitious RGA in a hybrid from BC’ × ‘M9′. The y-axis represents the G’ value, and the x-axis represents the physical position on the chromosome. Red lines represent ‘M9’, blue lines represent ‘BC’, and black lines represent ‘M9’ & ‘BC’.

**Additional file 3: Fig. S3** Differentially expressed unigenes (DEGs) in the extreme small and large RGA bulks in a hybrid from ‘BC’ × ‘M9’. (A) Statistical data of DEGs. (B-E) Kyoto Encyclopedia of Genes and Genomes (KEGG) pathway results for including plant hormone signalling (B), starch and sucrose metabolism (C), terpenoid backbone biosynthesis (D), and alpha-linolenic acid metabolism (E).

**Additional file 4: Fig. S4** Gene ontology (GO) analysis of differentially expressed genes (DEGs) from the RNA sequencing (RNA-seq) analysis. (A) GO classification. (B) GO enrichment. (C) GO statistics.

**Additional file 5: Fig. S5** Kyoto Encyclopedia of Genes and Genomes (KEGG) analysis of RNA-seq results. (A) KEGG classification. (B) KEGG statistics. (C) KEGG enrichment. (D) KEGG screening.

**Additional file 6: Fig. S6** Validation of *35S*:MdLAZY1 (A) and *35S*:MdNPR5 (B) transgenic *Nicotiana benthamiana* lines at the DNA (left) and cDNA (right) levels.

**Additional file 7: Fig. S7** Bimolecular fluorescence complementation (BiFC) assay showing protein-protein interactions. (A) MdLAZY1 and MdIAA1. (B) MdDREB2A and MdHSFB3. *BD-*DREB2A-BC (p) indicates a point mutation of C (SNP592) to T in MdDREB2A-BC. (C) MdLBD41 and MdbHLH48. Scale bars = 50 mm.

**Additional file 8: Table S1** The phenotype data for the adventitious root growth angle (RGA) of leafy cuttings of hybrids derived from ‘Baleng Crab (BC)’ (*Malus robusta*) × ‘M9’ (*M. pumila*) in 2016 and 2017. **Table S2** Summary of the results of bulked segregant analysis by next generation genomic sequencing (BSA-seq) of extreme bulks of small and large adventitious RGAs using hybrids derived from ‘BC’ × ‘M9’. **Table S3** Summary of BSA-seq detected QTLs for apple adventitious RGA before and after narrowing by changing slide window sizes. **Table S4** Twenty-four stem tissue samples for RNA sequencing (RNA-seq) taken from leafy cuttings of hybrids derived from ‘BC’ × ‘M9’. **Table S5** Summary of the sequencing reads and reads mapped with RNA-seq. **Table S6** Sample correlation analysis of three biological replicates with RNA-seq. **Table S7** KEGG pathway analysis of DEGs (ko00500, ko00592, ko00900, and ko04075) from RNA-seq results. **Table S8** Co-expression network of RGA identified by AppleMDO in hybrids from ‘BC’ × ‘M9’. **Table S9** Candidate genes for RGA screened by parental resequencing, BSA-seq, and RNA-seq in a hybrid population from ‘BC’ × ‘M9’. **Table S10** Candidate gene mining from BSA-seq regions based on multiomics data. **Table S11** Allelic variations of *MdLAZY1*, *MdIAA1*, *MdDREB2A*, *MdHSFB3*, *MdNPR5*, *MdLBD41*, and *MdbHLH48*. **Table S12** Closely linked relationship between six diagnostic markers (SNP-1485/− 474 of *MdLAZY1*, SNP223 of *MdIAA1*, SNP-1228 of *MdNPR5*, SNP908 of *MdLBD41*, and Del360 of *MdbHLH48*) and Kompetitive Allele Specific PCR (KASP) markers (b13, G122, S1272, Z312, and S1288). **Table S13** Joint genotype effects of markers linked to b13, SNP592, and G122 on RGA phenotype in hybrids from ‘BC’ × ‘M9’. **Table S14** Joint genotype effects of markers linked to S1272, Z312, and S1288 on RGA phenotype in hybrids from ‘BC’ × ‘M9’. **Table S15** Detailed data and primers for 26 allelic variations used for KASP in ‘BC’ and ‘M9’. **Table S16** The genotypes of 26 markers in 266 F1 hybrids from ‘BC’ × ‘M9’. **Table S17** The individual genotype effects of the 26 markers in 266 F1 hybrids from ‘BC’ × ‘M9’. **Table S18** Primers used for experimental validation in this study.

**Additional file 9: Supplementary File 1** Sequence alignment of *MdLAZY1* cloned from apple rootstocks ‘Baleng Crab (BC)’ (*Malus robusta*) and ‘M9’ (*M. pumila*). (A) Coding sequence (CDS). (B) Amino acid. C: Upstream.

**Additional file 10: Supplementary File 2** Sequence alignment of *MdIAA1*, *MdDREB2A*, and *MdHSFB3* cloned from apple rootstocks ‘BC’ and ‘M9’. (A), (C), and (E) CDS alignment of *MdIAA1* (A), *MdDREB2A* (C), and *MdHSFB3* (E), respectively. (B), (D), and (F) Amino acid sequences of *MdIAA1* (B), *MdDREB2A* (D), and *MdHSFB3* (F), respectively.

**Additional file 11: Supplementary File 3** Sequence alignment of *MdNPR5* cloned from apple rootstocks ‘BC’ and ‘M9’. (A) CDS. (B) Amino acid. (C) Upstream.

**Additional file 12: Supplementary File 4** Sequence alignment of *MdLBD41* cloned from apple rootstocks ‘BC’ and ‘M9’. (A) CDS. (B) Amino acid. (C) Upstream.

**Additional file 13: Supplementary File 5** Sequence alignment of *MdbHLH48* cloned from apple rootstocks ‘BC’ and ‘M9’. (A) CDS. (B) Amino acid. (C) Upstream.

## Data Availability

All RNA-seq reads and BSA-seq data have been deposited in the NCBI SRA under the accession number PRJNA655783 (https://www.ncbi.nlm.nih.gov/search/all/?term=PRJNA655783). The DNA resequencing reads of ‘Baleng Crab’ and ‘M9’ are freely available in the NCBI SRA under the accession numbers SRR12234087 (https://www.ncbi.nlm.nih.gov/search/all/?term=SRR12234087) and SRR12233711 (https://www.ncbi.nlm.nih.gov/search/all/?term=SRR12233711), respectively. The apple genome used was a version of the *Malus* × *domestica* genome GDDH13_v1.1 (GDDH13, https://iris.angers.inra.fr/gddh13/).

## Abbreviations

BiFC: Bimolecular fluorescence complementation
BSA-seq: bulked segregant analysis by sequencing
DEG: Differentially expressed unigene
KEGG: Kyoto Encyclopedia of Genes and Genomes
FPKM: Fragments per kilobase per million mapped reads
GO: Gene ontology
GS: Genomic selection
KASP: Kompetitive allele specific PCR
LUC: Luciferase
MAS: Marker-assisted selection
QTL: Quantitative trait locus
RGA: Root growth angle
RNA-seq: RNA sequencing
SNP: Single nucleotide polymorphism
WT: Wild type
Y1H: Yeast one-hybrid
Y2H: Yeast two-hybrid
