# Supplementary figures and images for: Intricate genetic variation networks control the adventitious root growth angle in apple
Source: BMC Genomics. 2020 Dec 1;21:852. doi: 10.1186/s12864-020-07257-8 (PMC7709433; doi:10.1186/s12864-020-07257-8)

A

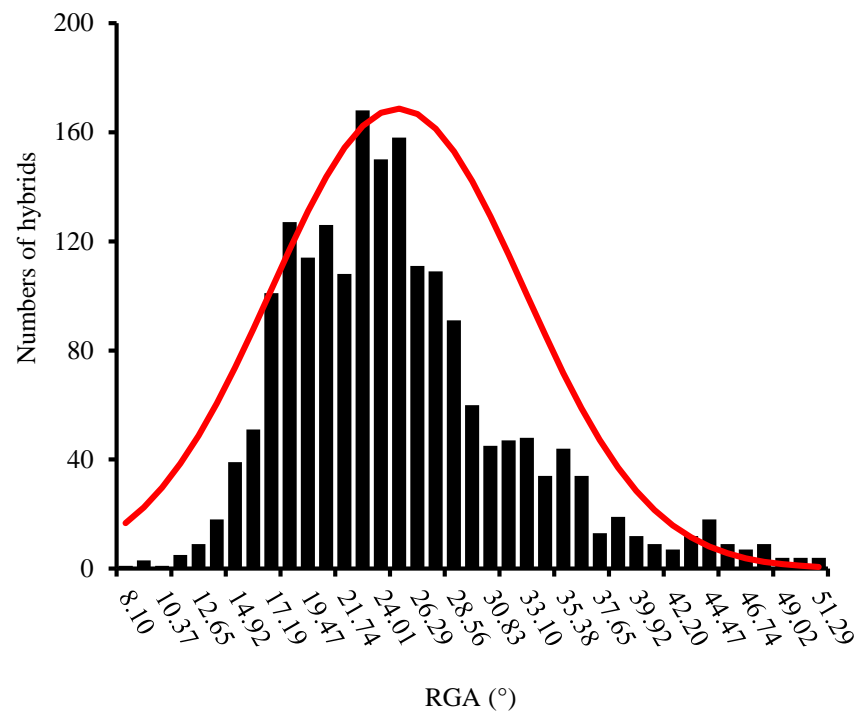

C

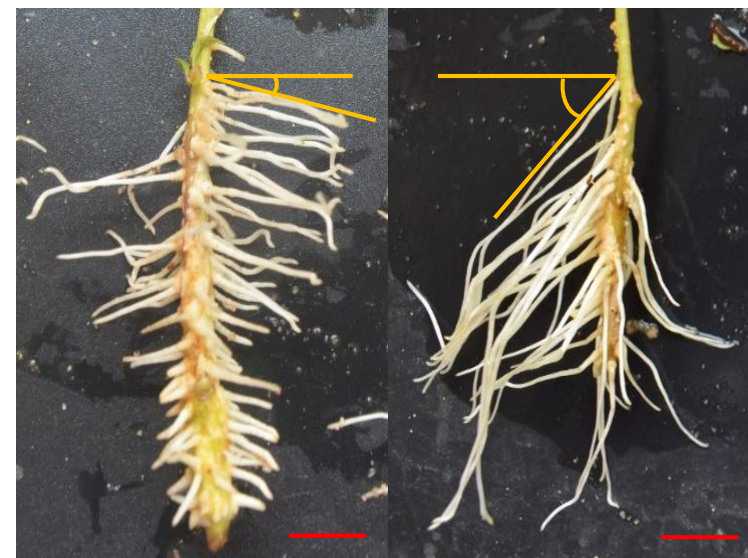

Small RGA (12.67°)

Large RGA (54.37°)

B

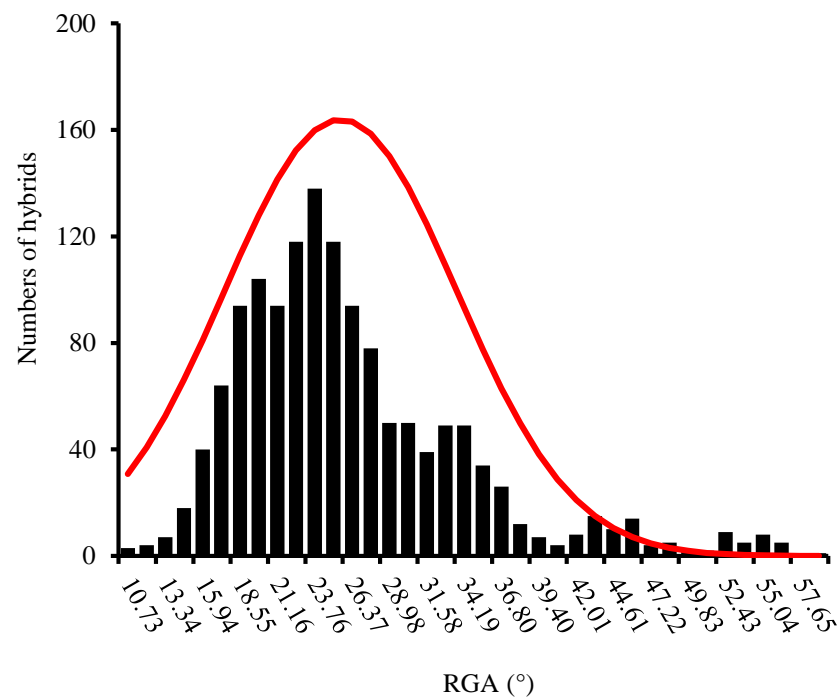

D

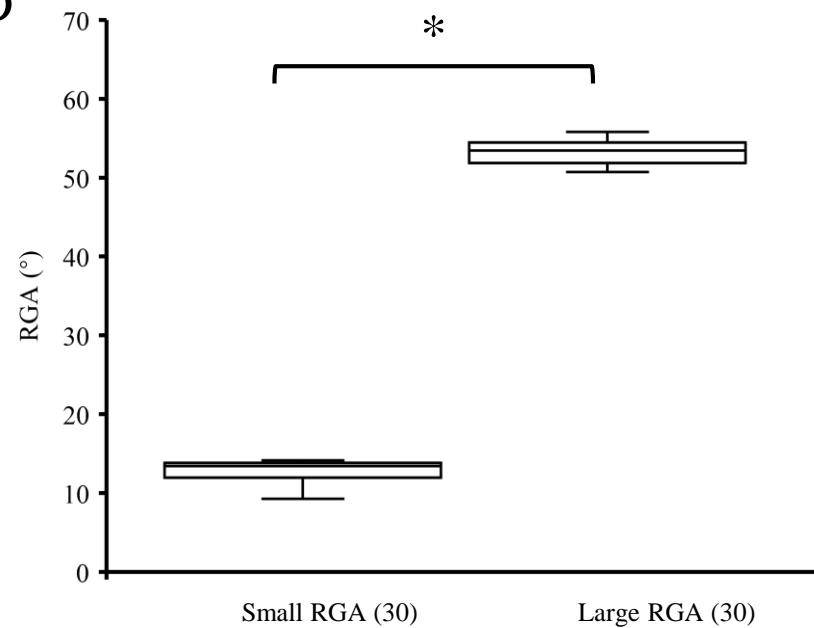

Supplement: Supplementary file 1 — Additional file 1: Fig. S1 Segregation and segregant bulk construction of the adventitious root growth angle (RGA) in leafy cuttings of a hybrid population derived from ‘Baleng Crab (BC)’ (Malus robusta) × ‘M9’ (M. pumila) in 2016 and 2017. (A) and (B) Frequency distribution diagrams of RGA in 2016 (A) and 2017 (B). (C) Photographs of adventitious roots in 30-day-old leafy cuttings, showing small and large RGAs. Scale bar = 10 mm. (D) Box plots showing the RGA phenotype in the small (left) and large (right) segregant bulks. Numbers of hybrids are presented in parentheses following the genotypes below the x-axis. Error bars indicate the standard deviation, and asterisks represent P < 0.05 by Dunnett’s multiple comparison. [file 12864_2020_7257_MOESM1_ESM.pdf]

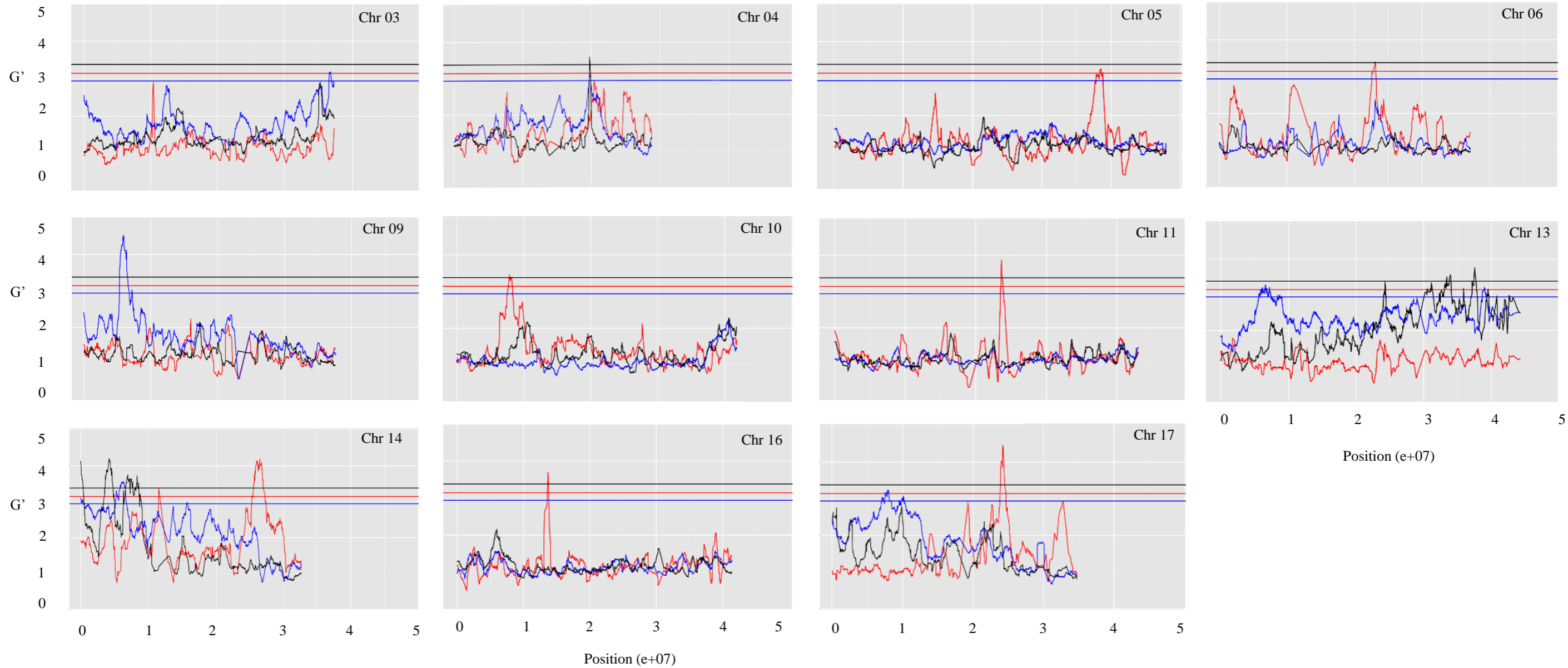

Supplement: Supplementary file 2 — Additional file 2: Fig. S2 Profiles of the significant quantitative trait loci (QTLs) for adventitious RGA in a hybrid from BC’ × ‘M9′. The y-axis represents the G’ value, and the x-axis represents the physical position on the chromosome. Red lines represent ‘M9’, blue lines represent ‘BC’, and black lines represent ‘M9’ & ‘BC’. [file 12864_2020_7257_MOESM2_ESM.pdf]

A

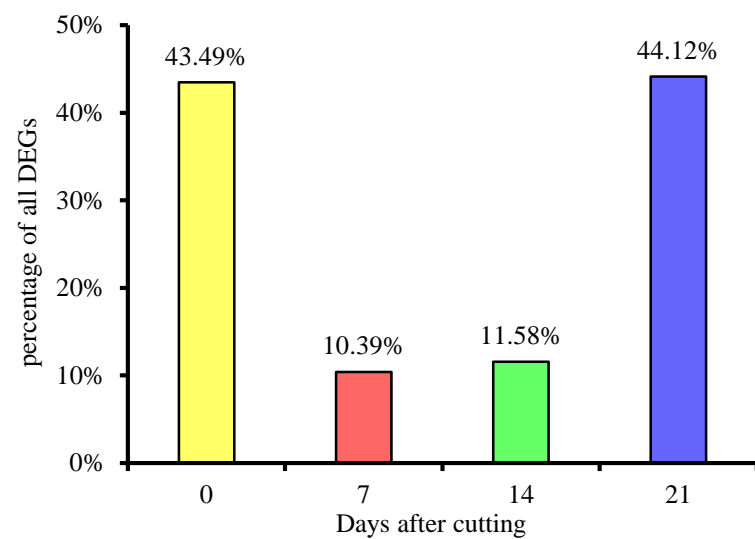

B

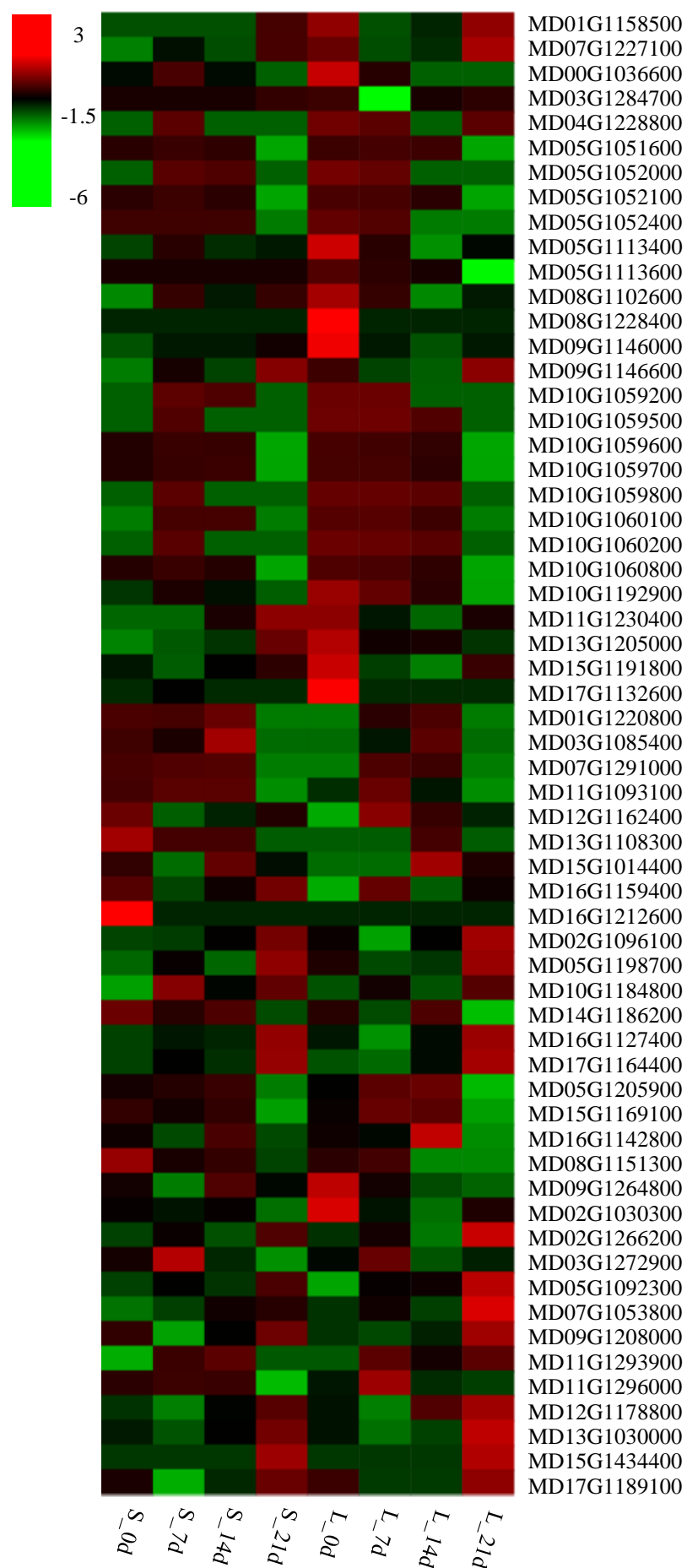

C

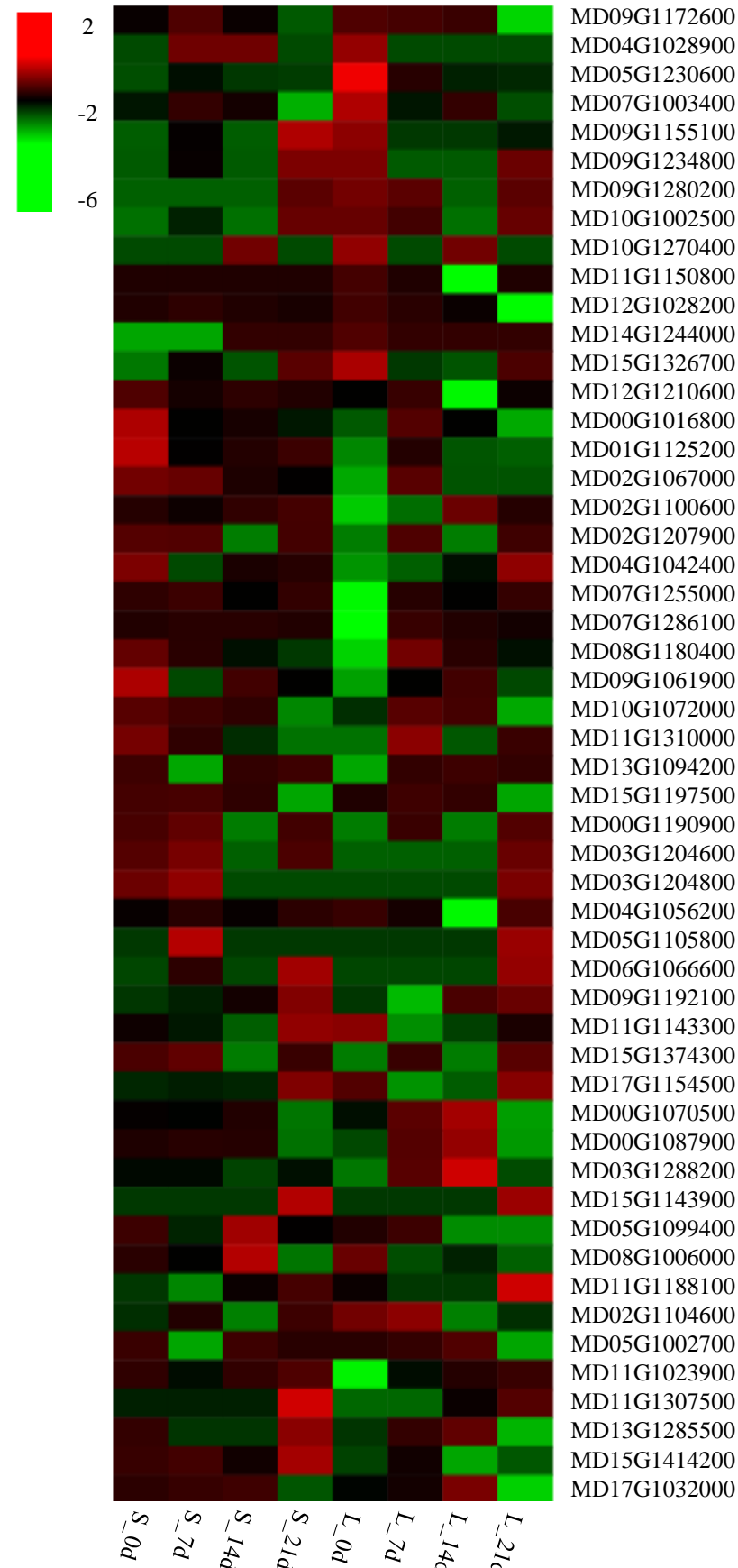

D

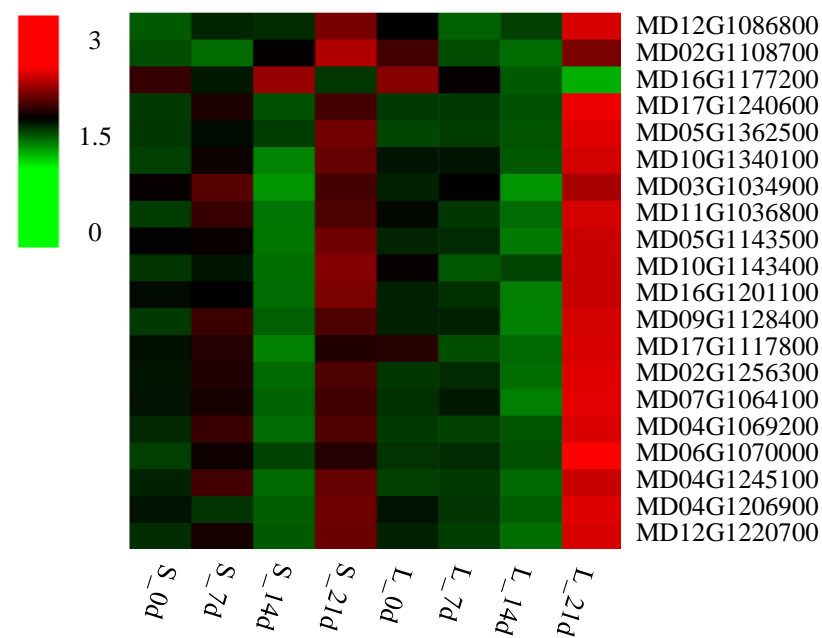

E

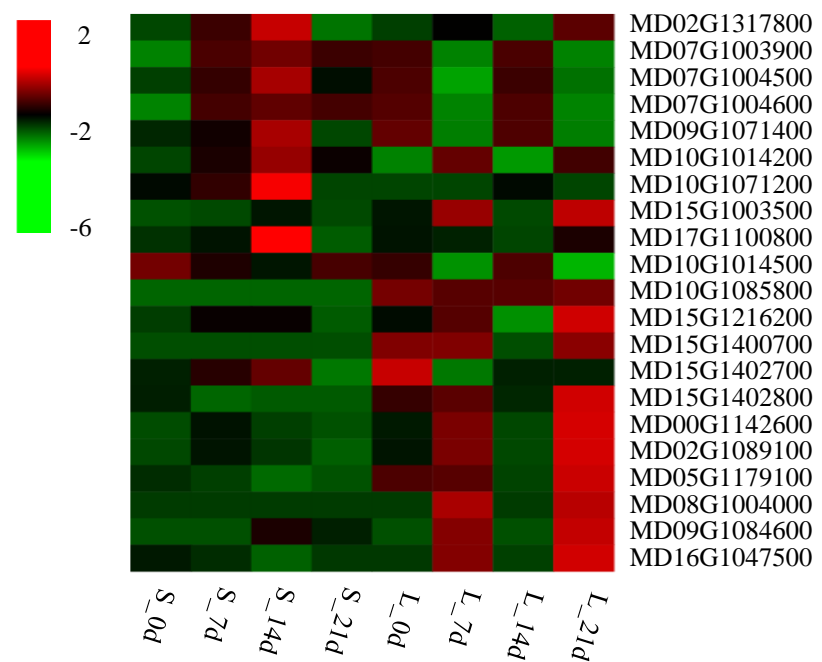

Supplement: Supplementary file 3 — Additional file 3: Fig. S3 Differentially expressed unigenes (DEGs) in the extreme small and large RGA bulks in a hybrid from ‘BC’ × ‘M9’. (A) Statistical data of DEGs. (B-E) Kyoto Encyclopedia of Genes and Genomes (KEGG) pathway results for including plant hormone signalling (B), starch and sucrose metabolism (C), terpenoid backbone biosynthesis (D), and alpha-linolenic acid metabolism (E). [file 12864_2020_7257_MOESM3_ESM.pdf]

A

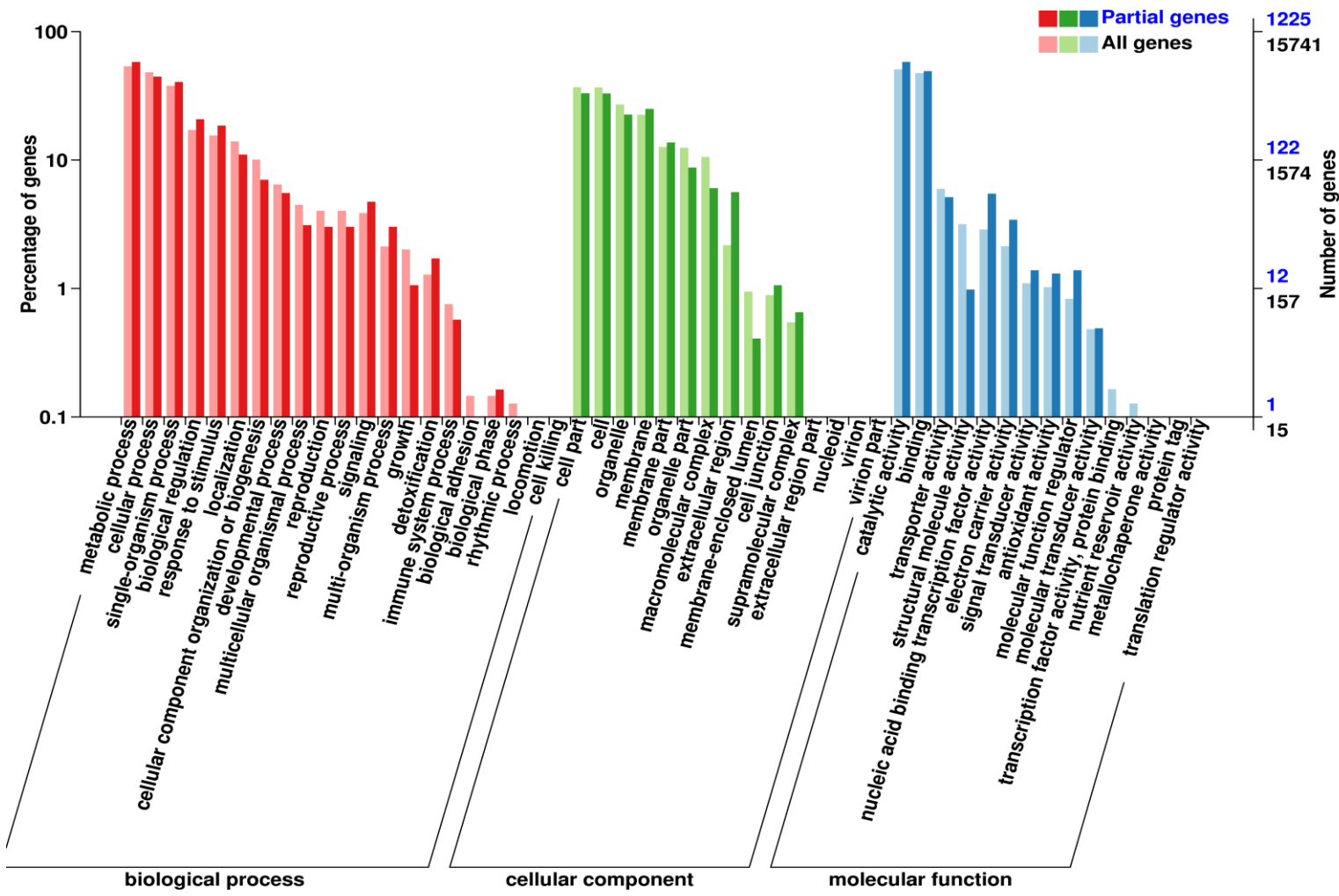

B

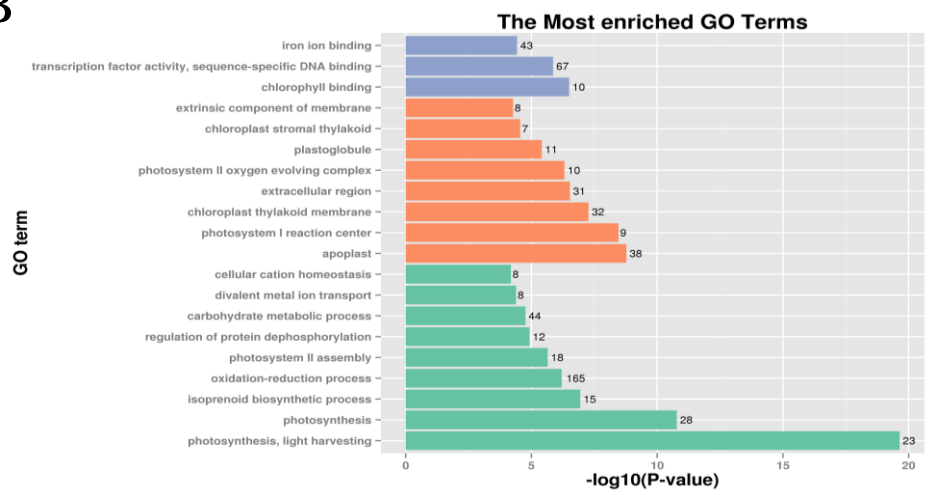

C

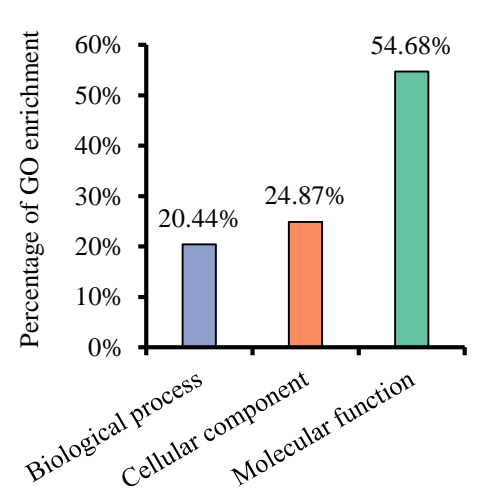

Supplement: Supplementary file 4 — Additional file 4: Fig. S4 Gene ontology (GO) analysis of differentially expressed genes (DEGs) from the RNA sequencing (RNA-seq) analysis. (A) GO classification. (B) GO enrichment. (C) GO statistics. [file 12864_2020_7257_MOESM4_ESM.pdf]

A

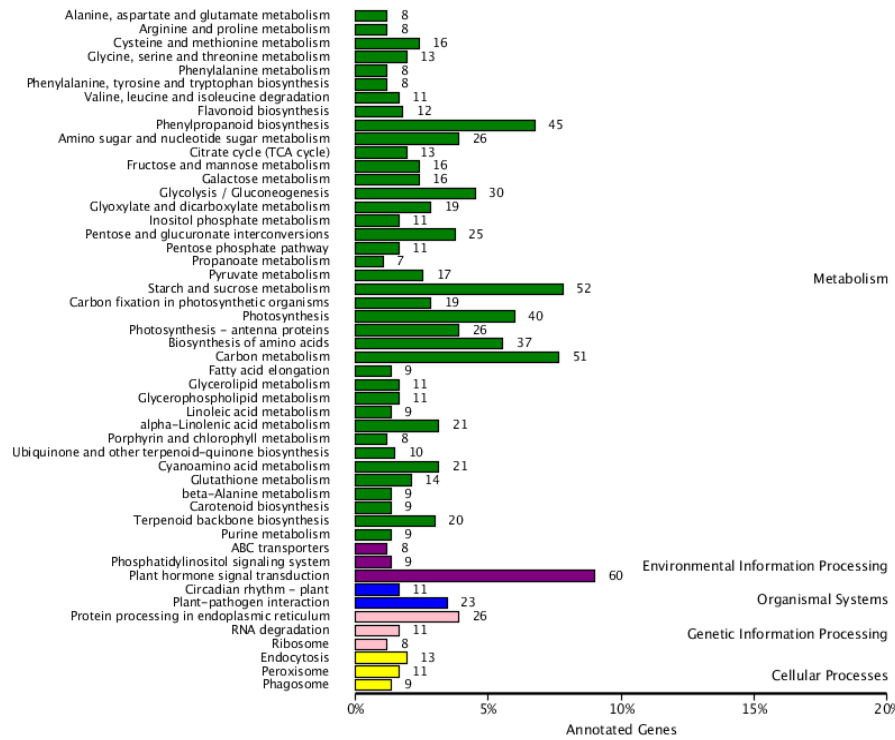

B

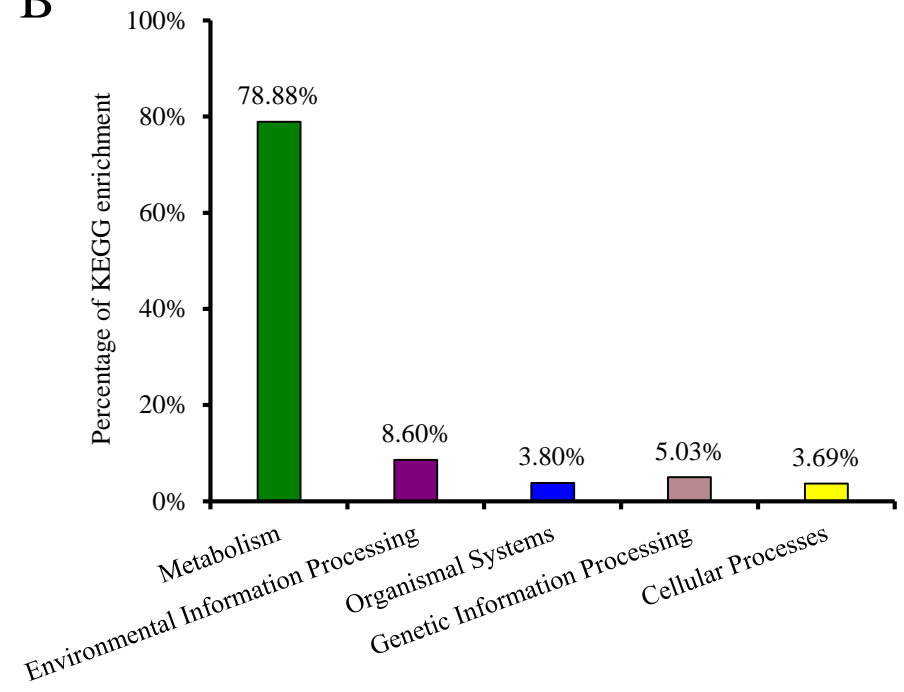

C

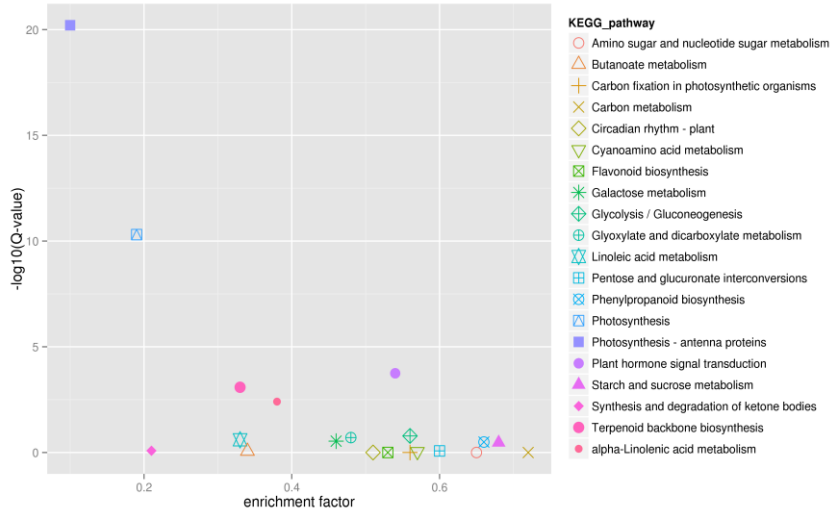

D

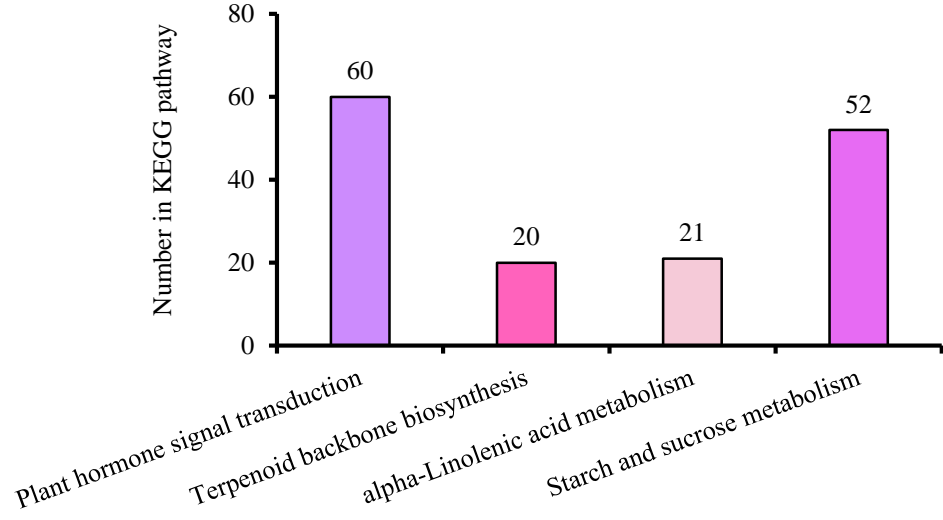

Supplement: Supplementary file 5 — Additional file 5: Fig. S5 Kyoto Encyclopedia of Genes and Genomes (KEGG) analysis of RNA-seq results. (A) KEGG classification. (B) KEGG statistics. (C) KEGG enrichment. (D) KEGG screening. [file 12864_2020_7257_MOESM5_ESM.pdf]

**A**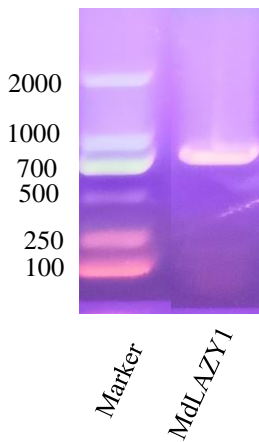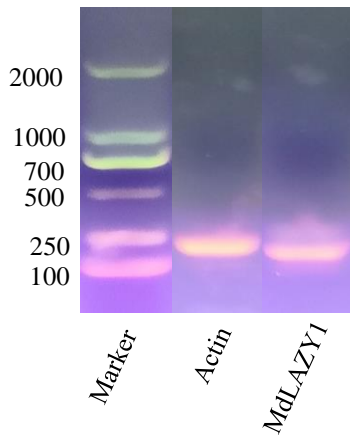**B**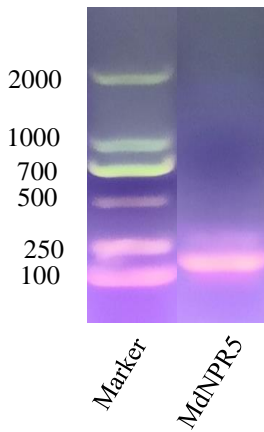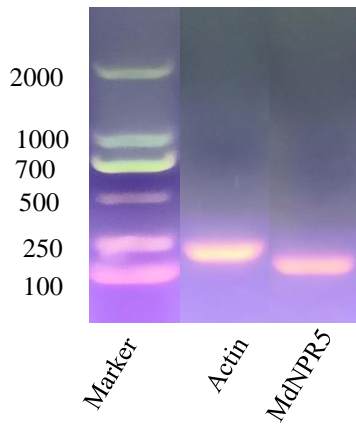

Supplement: Supplementary file 6 — Additional file 6: Fig. S6 Validation of 35S:MdLAZY1 (A) and 35S:MdNPR5 (B) transgenic Nicotiana benthamiana lines at the DNA (left) and cDNA (right) levels. [file 12864_2020_7257_MOESM6_ESM.pdf]

A

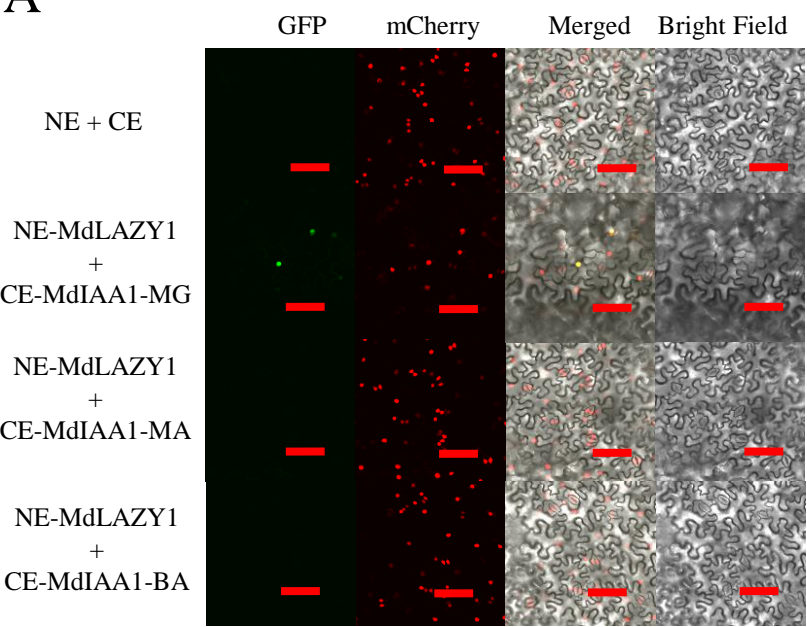

B

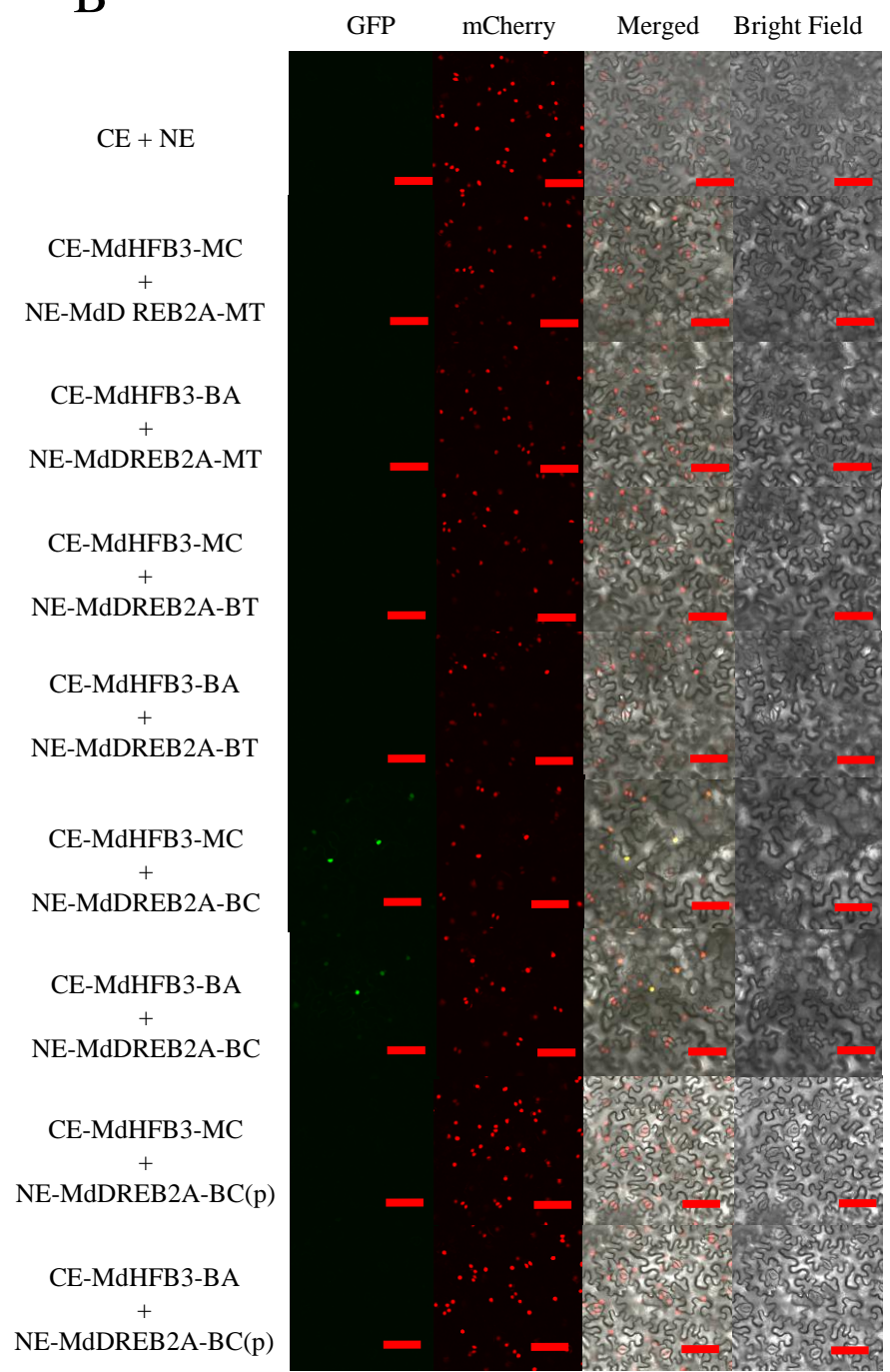

C

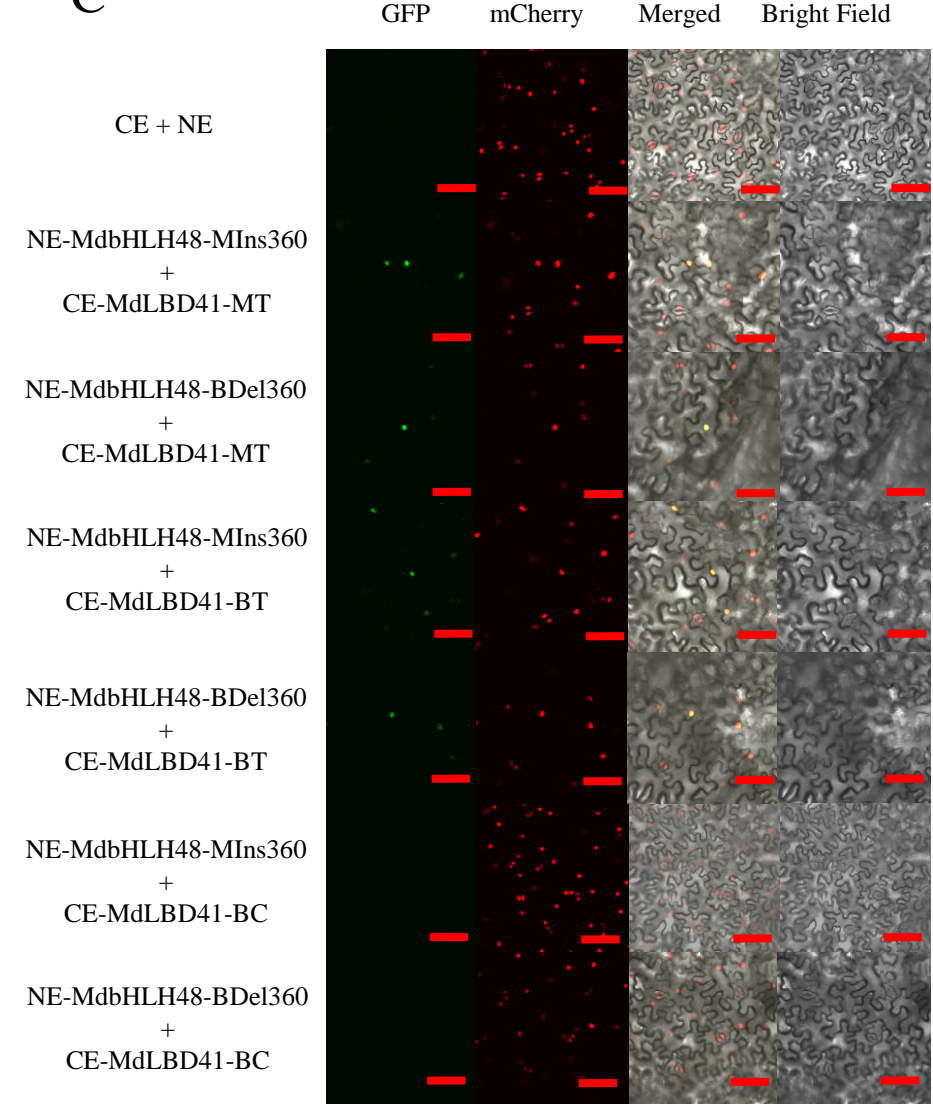

Supplement: Supplementary file 7 — Additional file 7: Fig. S7 Bimolecular fluorescence complementation (BiFC) assay showing protein-protein interactions. (A) MdLAZY1 and MdIAA1. (B) MdDREB2A and MdHSFB3. BD-DREB2A-BC (p) indicates a point mutation of C (SNP592) to T in MdDREB2A-BC. (C) MdLBD41 and MdbHLH48. Scale bars = 50 mm. [file 12864_2020_7257_MOESM7_ESM.pdf]
