# Supplementary material for: Intricate genetic variation networks control the adventitious root growth angle in apple
Source: BMC Genomics. 2020 Dec 1;21:852. doi: 10.1186/s12864-020-07257-8 (PMC7709433; doi:10.1186/s12864-020-07257-8)
Supplement: Supplementary file 10 — Additional file 10: Supplementary File 2 Sequence alignment of MdIAA1, MdDREB2A, and MdHSFB3 cloned from apple rootstocks ‘BC’ and ‘M9’. (A), (C), and (E) CDS alignment of MdIAA1 (A), MdDREB2A (C), and MdHSFB3 (E), respectively. (B), (D), and (F) Amino acid sequences of MdIAA1 (B), MdDREB2A (D), and MdHSFB3 (F), respectively. [file 12864_2020_7257_MOESM10_ESM.pdf]

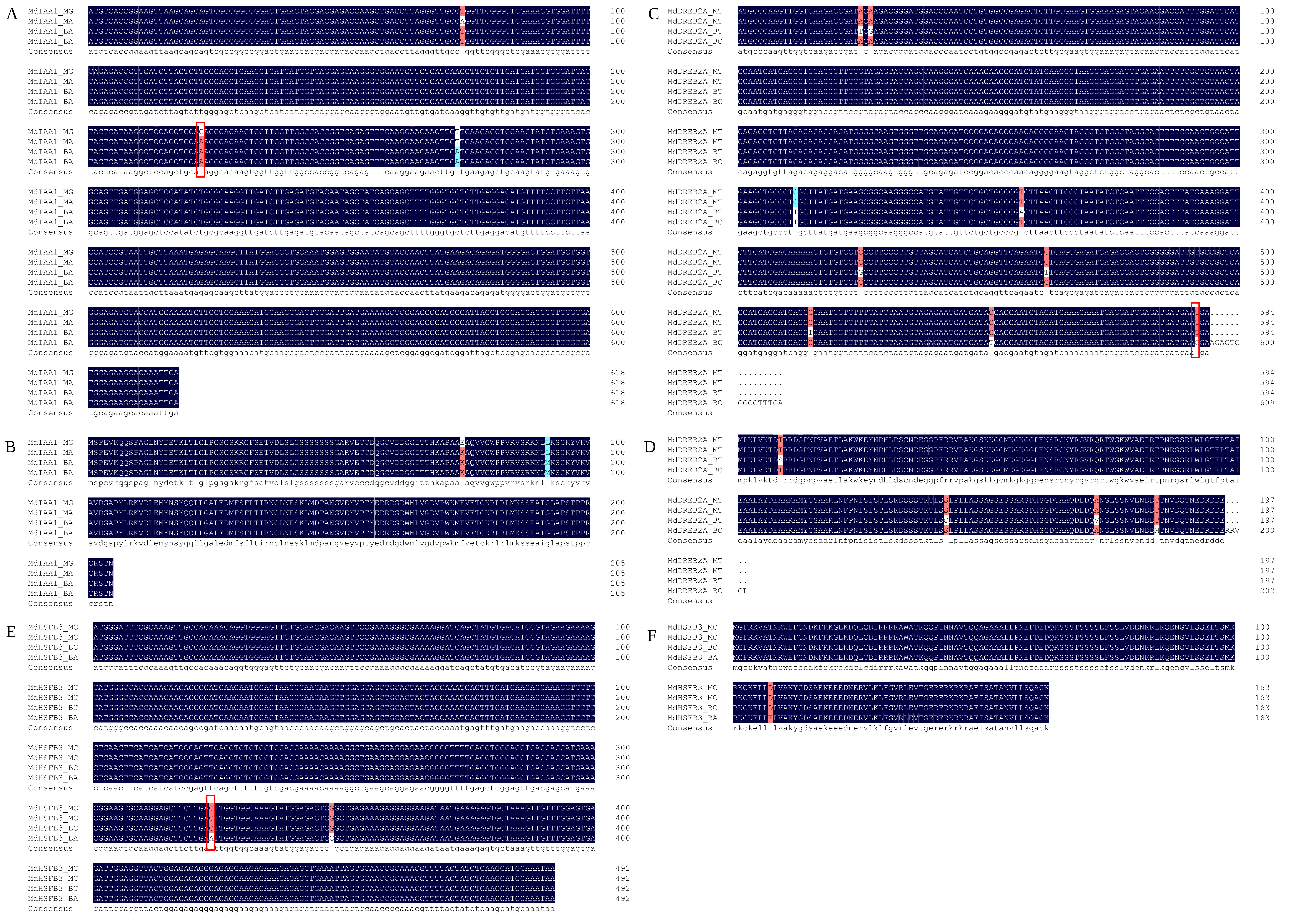
A
MdIAA1_MG
MdIAA1_MA
MdIAA1_BA
Consensus
B
C
MdDREB2A_MT
MdDREB2A_BT
MdDREB2A_BC
Consensus
D
E
MdHSFB3_MC
MdHSFB3_BC
MdHSFB3_BA
Consensus
F
