# Supplementary material for: Intricate genetic variation networks control the adventitious root growth angle in apple
Source: BMC Genomics. 2020 Dec 1;21:852. doi: 10.1186/s12864-020-07257-8 (PMC7709433; doi:10.1186/s12864-020-07257-8)
Supplement: Supplementary file 11 — Additional file 11: Supplementary File 3 Sequence alignment of MdNPR5 cloned from apple rootstocks ‘BC’ and ‘M9’. (A) CDS. (B) Amino acid. (C) Upstream. [file 12864_2020_7257_MOESM11_ESM.pdf]

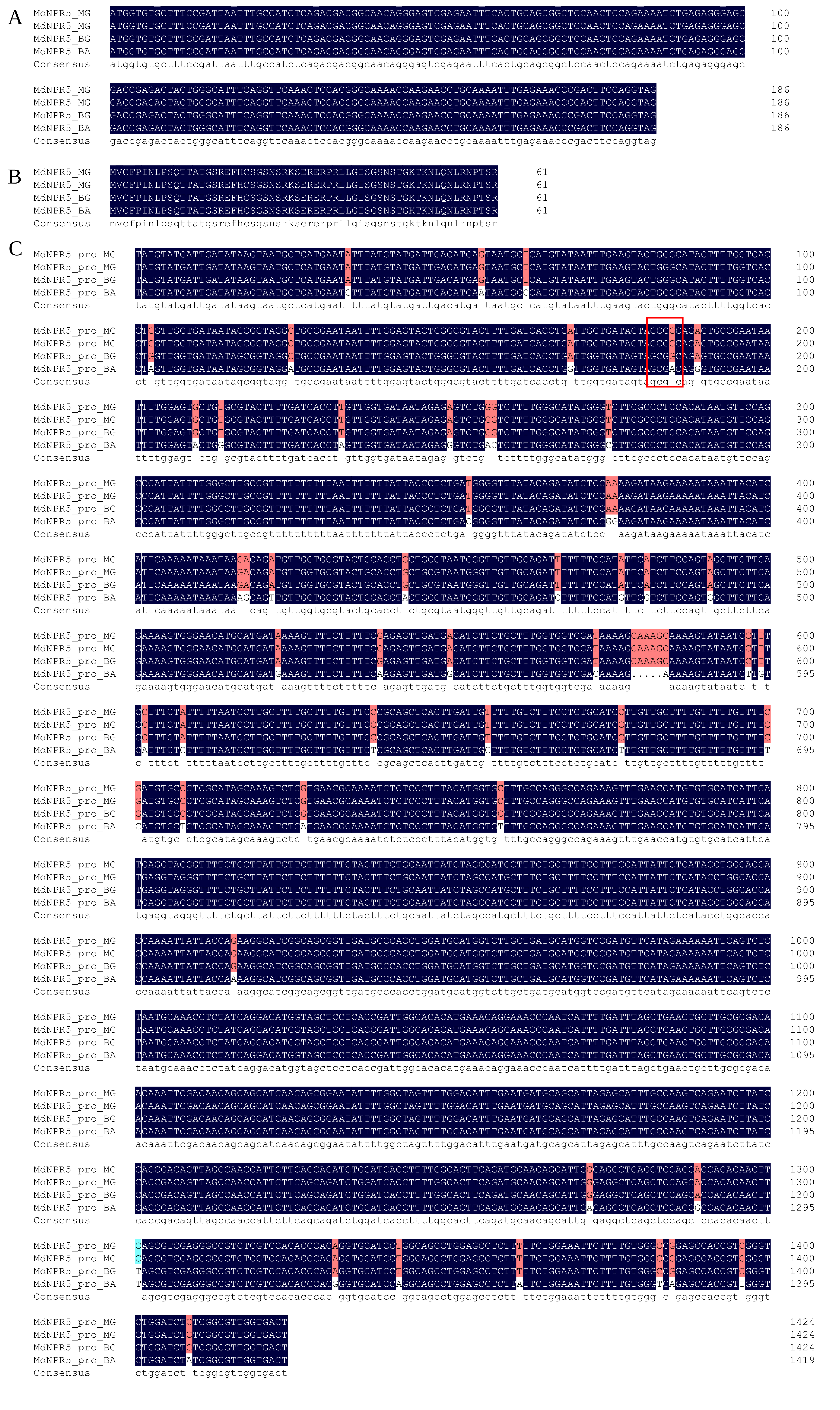
A
MdNPR5_MG ATGGTGTGCTTTCCGATTAATTTGCCATCTCAGACGACGGCAACAGGGAGTCGAGAATTTCACTGCAGCGGCTCCAACTCCAGAAAATCTGAGAGGGAGC 100
MdNPR5_MG ATGGTGTGCTTTCCGATTAATTTGCCATCTCAGACGACGGCAACAGGGAGTCGAGAATTTCACTGCAGCGGCTCCAACTCCAGAAAATCTGAGAGGGAGC 100
MdNPR5_BG ATGGTGTGCTTTCCGATTAATTTGCCATCTCAGACGACGGCAACAGGGAGTCGAGAATTTCACTGCAGCGGCTCCAACTCCAGAAAATCTGAGAGGGAGC 100
MdNPR5_BA ATGGTGTGCTTTCCGATTAATTTGCCATCTCAGACGACGGCAACAGGGAGTCGAGAATTTCACTGCAGCGGCTCCAACTCCAGAAAATCTGAGAGGGAGC 100
Consensus atggtgtgctttccgattaatttgccatctcagacgacggcaacagggagtcgagaatttcactgcagcggctccaactccagaaaatctgagagggagc
MdNPR5_MG GACCGAGACTACTGGGCATTTCAGGTTCAAACTCCACGGGCAAAACCAAGAACCTGCAAAATTTGAGAAACCCGACTTCCAGGTAG 186
MdNPR5_MG GACCGAGACTACTGGGCATTTCAGGTTCAAACTCCACGGGCAAAACCAAGAACCTGCAAAATTTGAGAAACCCGACTTCCAGGTAG 186
MdNPR5_BG GACCGAGACTACTGGGCATTTCAGGTTCAAACTCCACGGGCAAAACCAAGAACCTGCAAAATTTGAGAAACCCGACTTCCAGGTAG 186
MdNPR5_BA GACCGAGACTACTGGGCATTTCAGGTTCAAACTCCACGGGCAAAACCAAGAACCTGCAAAATTTGAGAAACCCGACTTCCAGGTAG 186
Consensus gaccgagactactgggcatttcaggttcaaactccacgggcaaaaccaagaacctgcaaaatttgagaaacccgacttccaggtag
B
MdNPR5_MG MVCFPINLPSQTTATGSREFHCSGSNSRKSERERPRLLGISGSNSTGKTKNLQNLRNPTSR 61
MdNPR5_MG MVCFPINLPSQTTATGSREFHCSGSNSRKSERERPRLLGISGSNSTGKTKNLQNLRNPTSR 61
MdNPR5_BG MVCFPINLPSQTTATGSREFHCSGSNSRKSERERPRLLGISGSNSTGKTKNLQNLRNPTSR 61
MdNPR5_BA MVCFPINLPSQTTATGSREFHCSGSNSRKSERERPRLLGISGSNSTGKTKNLQNLRNPTSR 61
Consensus mvcfpinlpsqttatgsrefhcsgsnsrksererprllgisgsnstgktknlqnlrnptsr
C
MdNPR5_pro_MG TATGTATGATTGATATAAGTAATGCTCATGAATATTTATGTATGATTGACATGAGTAATGCTCATGTATAATTTGAAGTACTGGGCATACTTTTGGTCAC 100
MdNPR5_pro_MG TATGTATGATTGATATAAGTAATGCTCATGAATATTTATGTATGATTGACATGAGTAATGCTCATGTATAATTTGAAGTACTGGGCATACTTTTGGTCAC 100
MdNPR5_pro_BG TATGTATGATTGATATAAGTAATGCTCATGAATATTTATGTATGATTGACATGAGTAATGCTCATGTATAATTTGAAGTACTGGGCATACTTTTGGTCAC 100
MdNPR5_pro_BA TATGTATGATTGATATAAGTAATGCTCATGAATGTTTATGTATGATTGACATGAATAATGCCCATGTATAATTTGAAGTACTGGGCATACTTTTGGTCAC 100
Consensus tatgtatgattgatataagtaatgctcatgaat tttatgtatgattgacatga taatgc catgtataatttgaagtactgggcatacttttggtcac
MdNPR5_pro_MG CTGGTTGGTGATAATAGCGGTAGGCTGCCGAATAATTTTGGAGTACTGGGCGTACTTTTGATCACCTGATTGGTGATAGTAGCGGCAGAGTGCCGAATAA 200
MdNPR5_pro_MG CTGGTTGGTGATAATAGCGGTAGGCTGCCGAATAATTTTGGAGTACTGGGCGTACTTTTGATCACCTGATTGGTGATAGTAGCGGCAGAGTGCCGAATAA 200
MdNPR5_pro_BG CTGGTTGGTGATAATAGCGGTAGGCTGCCGAATAATTTTGGAGTACTGGGCGTACTTTTGATCACCTGATTGGTGATAGTAGCGGCAGAGTGCCGAATAA 200
MdNPR5_pro_BA CTAGTTGGTGATAATAGCGGTAGGATGCCGAATAATTTTGGAGTACTGGGCGTACTTTTGATCACCTGGTTGGTGATAGTAGCGACAGGGTGCCGAATAA 200
Consensus ct gttggtgataatagcggtagg tgccgaataattttggagtactgggcgtacttttgatcacctg ttggtgatagtagcg cag gtgccgaataa
MdNPR5_pro_MG TTTTGGAGTGCTGTGCGTACTTTTGATCACCTTGTTGGTGATAATAGAGAGTCTGGGTCTTTTGGGCATATGGGTCTTCGCCCTCCACATAATGTTCCAG 300
MdNPR5_pro_MG TTTTGGAGTGCTGTGCGTACTTTTGATCACCTTGTTGGTGATAATAGAGAGTCTGGGTCTTTTGGGCATATGGGTCTTCGCCCTCCACATAATGTTCCAG 300
MdNPR5_pro_BG TTTTGGAGTGCTGTGCGTACTTTTGATCACCTTGTTGGTGATAATAGAGAGTCTGGGTCTTTTGGGCATATGGGTCTTCGCCCTCCACATAATGTTCCAG 300
MdNPR5_pro_BA TTTTGGAGTACTGGGCGTACTTTTGATCACCTAGTTGGTGATAATAGAGGGTCTGACTCTTTTGGGCATATGGGCCTTCGCCCTCCACATAATGTTCCAG 300
Consensus ttttggagt ctg gcgtacttttgatcacct gttggtgataatagag gtctg tcttttgggcatatggg cttcgccctccacataatgttccag
MdNPR5_pro_MG CCCATTATTTTGGGCTTGCCGTTTTTTTTTAATTTTTTTATTACCCTCTGATGGGGTTTATACAGATATCTCCAAAAGATAAGAAAAATAAATTACATC 400
MdNPR5_pro_MG CCCATTATTTTGGGCTTGCCGTTTTTTTTTAATTTTTTTATTACCCTCTGATGGGGTTTATACAGATATCTCCAAAAGATAAGAAAAATAAATTACATC 400
MdNPR5_pro_BG CCCATTATTTTGGGCTTGCCGTTTTTTTTTAATTTTTTTATTACCCTCTGATGGGGTTTATACAGATATCTCCAAAAGATAAGAAAAATAAATTACATC 400
MdNPR5_pro_BA CCCATTATTTTGGGCTTGCCGTTTTTTTTTAATTTTTTTATTACCCTCTGACGGGGTTTATACAGATATCTCCGGAAGATAAGAAAAATAAATTACATC 400
Consensus cccattattttgggcttgccgtttttttttaatttttttattaccctctga ggggtttatacagatatctcc aagataagaaaaataaattacatc
MdNPR5_pro_MG ATTCAAAAATAAATAAGACAGATGTTGGTGCGTACTGCACCTGCTGCGTAATGGGTTGTTGCAGATTTTTTTCCATATTCATCTTCCAGTAGCTTCTTCA 500
MdNPR5_pro_MG ATTCAAAAATAAATAAGACAGATGTTGGTGCGTACTGCACCTGCTGCGTAATGGGTTGTTGCAGATTTTTTTCCATATTCATCTTCCAGTAGCTTCTTCA 500
MdNPR5_pro_BG ATTCAAAAATAAATAAGACAGATGTTGGTGCGTACTGCACCTGCTGCGTAATGGGTTGTTGCAGATTTTTTTCCATATTCATCTTCCAGTAGCTTCTTCA 500
MdNPR5_pro_BA ATTCAAAAATAAATAAAGCAGTTGTTGGTGCGTACTGCACCTACTGCGTAATGGGTTGTTGCAGATCTTTTTCCATGTTCGTCTTCCAGTGGCTTCTTCA 500
Consensus attcaaaaataaataa cag tgttggtgcgtactgcacct ctgcgtaatgggttgttgcagat tttttccat ttc tcttccagt gcttcttca
MdNPR5_pro_MG GAAAAGTGGGAACATGCATGATAAAAGTTTTCTTTTTCGAGAGTTGATGACATCTTCTGCTTTGGTGGTCGATAAAAGCAAAGCAAAAGTATAATCCTTT 600
MdNPR5_pro_MG GAAAAGTGGGAACATGCATGATAAAAGTTTTCTTTTTCGAGAGTTGATGACATCTTCTGCTTTGGTGGTCGATAAAAGCAAAGCAAAAGTATAATCCTTT 600
MdNPR5_pro_BG GAAAAGTGGGAACATGCATGATAAAAGTTTTCTTTTTCGAGAGTTGATGACATCTTCTGCTTTGGTGGTCGATAAAAGCAAAGCAAAAGTATAATCCTTT 600
MdNPR5_pro_BA GAAAAGTGGGAACATGCATGATGAAAGTTTTCTTTTTCAAGAGTTGATGGCATCTTCTGCTTTGGTGGTCGACAAAAG.....AAAAAGTATAATCTTGT 595
Consensus gaaaagtgggaacatgcatgat aaagttttctttttc agagttgatg catcttctgctttggtggtcga aaaag   aaaagtataatc t t
MdNPR5_pro_MG CCTTTCTATTTTTAATCCTTGCTTTTGCTTTTGTTTCCCGCAGCTCACTTGATTGTTTTTGTCTTTCCTCTGCATCCTTGTTGCTTTTGTTTTTGTTTTC 700
MdNPR5_pro_MG CCTTTCTATTTTTAATCCTTGCTTTTGCTTTTGTTTCCCGCAGCTCACTTGATTGTTTTTGTCTTTCCTCTGCATCCTTGTTGCTTTTGTTTTTGTTTTC 700
MdNPR5_pro_BG CCTTTCTATTTTTAATCCTTGCTTTTGCTTTTGTTTCCCGCAGCTCACTTGATTGTTTTTGTCTTTCCTCTGCATCCTTGTTGCTTTTGTTTTTGTTTTC 700
MdNPR5_pro_BA CATTTCTCTTTTTAATCCTTGCTTTTGCTTTTGTTTCTCGCAGCTCACTTGATTGCTTTTGTCTTTCCTCTGCATCTTTGTTGCTTTTGTTTTTGTTTTT 695
Consensus c tttct tttttaatccttgcttttgcttttgtttc cgcagctcacttgattg ttttgtctttcctctgcatc ttgttgcttttgtttttgtttt
MdNPR5_pro_MG GATGTGCCCTCGCATAGCAAAGTCTCGTGAACGCAAAATCTCTCCCTTTACATGGTGCTTTGCCAGGGCCAGAAAGTTTGAACCATGTGTGCATCATTCA 800
MdNPR5_pro_MG GATGTGCCCTCGCATAGCAAAGTCTCGTGAACGCAAAATCTCTCCCTTTACATGGTGCTTTGCCAGGGCCAGAAAGTTTGAACCATGTGTGCATCATTCA 800
MdNPR5_pro_BG GATGTGCCCTCGCATAGCAAAGTCTCGTGAACGCAAAATCTCTCCCTTTACATGGTGCTTTGCCAGGGCCAGAAAGTTTGAACCATGTGTGCATCATTCA 800
MdNPR5_pro_BA CATGTGCTCTCGCATAGCAAAGTCTCATGAACGCAAAATCTCTCCCTTTACATGGTGTTTTGCCAGGGCCAGAAAGTTTGAACCATGTGTGCATCATTCA 795
Consensus atgtgc ctcgcatagcaaagtctc tgaacgcaaaatctctccctttacatggtg tttgccagggccagaaagtttgaaccatgtgtgcatcattca
MdNPR5_pro_MG TGAGGTAGGGTTTTCTGCTTATTCTTCTTTTTTCTACTTTCTGCAATTATCTAGCCATGCTTTCTGCTTTTCCTTTCCATTATTCTCATACCTGGCACCA 900
MdNPR5_pro_MG TGAGGTAGGGTTTTCTGCTTATTCTTCTTTTTTCTACTTTCTGCAATTATCTAGCCATGCTTTCTGCTTTTCCTTTCCATTATTCTCATACCTGGCACCA 900
MdNPR5_pro_BG TGAGGTAGGGTTTTCTGCTTATTCTTCTTTTTTCTACTTTCTGCAATTATCTAGCCATGCTTTCTGCTTTTCCTTTCCATTATTCTCATACCTGGCACCA 900
MdNPR5_pro_BA TGAGGTAGGGTTTTCTGCTTATTCTTCTTTTTTCTACTTTCTGCAATTATCTAGCCATGCTTTCTGCTTTTCCTTTCCATTATTCTCATACCTGGCACCA 895
Consensus tgaggtagggttttctgcttattcttcttttttctactttctgcaattatctagccatgctttctgcttttcctttccattattctcatacctggcacca
MdNPR5_pro_MG CCAAAATTATTACCAGAAGGCATCGGCAGCGGTTGATGCCCACCTGGATGCATGGTCTTGCTGATGCATGGTCCGATGTTCATAGAAAAAAATTCAGTCTC 1000
MdNPR5_pro_MG CCAAAATTATTACCAGAAGGCATCGGCAGCGGTTGATGCCCACCTGGATGCATGGTCTTGCTGATGCATGGTCCGATGTTCATAGAAAAAAATTCAGTCTC 1000
MdNPR5_pro_BG CCAAAATTATTACCAGAAGGCATCGGCAGCGGTTGATGCCCACCTGGATGCATGGTCTTGCTGATGCATGGTCCGATGTTCATAGAAAAAAATTCAGTCTC 1000
MdNPR5_pro_BA CCAAAATTATTACCAAAAGGCATCGGCAGCGGTTGATGCCCACCTGGATGCATGGTCTTGCTGATGCATGGTCCGATGTTCATAGAAAAAAATTCAGTCTC 995
Consensus ccaaaattattacca aaggcatcggcagcggttgatgcccacctggatgcatggtcttgctgatgcatggtccgatgttcatagaaaaaaattcagtctc
MdNPR5_pro_MG TAATGCAAACCTCTATCAGGACATGGTAGCTCCTCACCGATTGGCACACATGAAACAGGAAACCCAATCATTTTGATTTAGCTGAACTGCTTGCGCGACA 1100
MdNPR5_pro_MG TAATGCAAACCTCTATCAGGACATGGTAGCTCCTCACCGATTGGCACACATGAAACAGGAAACCCAATCATTTTGATTTAGCTGAACTGCTTGCGCGACA 1100
MdNPR5_pro_BG TAATGCAAACCTCTATCAGGACATGGTAGCTCCTCACCGATTGGCACACATGAAACAGGAAACCCAATCATTTTGATTTAGCTGAACTGCTTGCGCGACA 1100
MdNPR5_pro_BA TAATGCAAACCTCTATCAGGACATGGTAGCTCCTCACCGATTGGCACACATGAAACAGGAAACCCAATCATTTTGATTTAGCTGAACTGCTTGCGCGACA 1095
Consensus taatgcaaacctctatcaggacatggtagctcctcaccgattggcacacatgaaacaggaaacccaatcattttgatttagctgaactgcttgcgcgaca
MdNPR5_pro_MG ACAAATTCGACAACAGCAGCATCAACAGCGGAATATTTTGGCTAGTTTTGGACATTTGAATGATGCAGCATTAGAGCATTTGCCAAGTCAGAATCTTATC 1200
MdNPR5_pro_MG ACAAATTCGACAACAGCAGCATCAACAGCGGAATATTTTGGCTAGTTTTGGACATTTGAATGATGCAGCATTAGAGCATTTGCCAAGTCAGAATCTTATC 1200
MdNPR5_pro_BG ACAAATTCGACAACAGCAGCATCAACAGCGGAATATTTTGGCTAGTTTTGGACATTTGAATGATGCAGCATTAGAGCATTTGCCAAGTCAGAATCTTATC 1200
MdNPR5_pro_BA ACAAATTCGACAACAGCAGCATCAACAGCGGAATATTTTGGCTAGTTTTGGACATTTGAATGATGCAGCATTAGAGCATTTGCCAAGTCAGAATCTTATC 1195
Consensus acaaattcgacaacagcagcatcaacagcggaatattttggctagttttggacatttgaatgatgcagcattagagcatttgccaagtcagaatcttatc
MdNPR5_pro_MG CACCGACAGTTAGCCAACCATTCTTCAGCAGATCTGGATCACCTTTTGGCACTTCAGATGCAACAGCATTGGGAGGCTCAGCTCCAGCACCACACAACTT 1300
MdNPR5_pro_MG CACCGACAGTTAGCCAACCATTCTTCAGCAGATCTGGATCACCTTTTGGCACTTCAGATGCAACAGCATTGGGAGGCTCAGCTCCAGCACCACACAACTT 1300
MdNPR5_pro_BG CACCGACAGTTAGCCAACCATTCTTCAGCAGATCTGGATCACCTTTTGGCACTTCAGATGCAACAGCATTGGGAGGCTCAGCTCCAGCACCACACAACTT 1300
MdNPR5_pro_BA CACCGACAGTTAGCCAACCATTCTTCAGCAGATCTGGATCACCTTTTGGCACTTCAGATGCAACAGCATTGAGAGGCTCAGCTCCAGCGCCACACAACTT 1295
Consensus caccgacagttagccaaccattcttcagcagatctggatcaccttttggcacttcagatgcaacagcattg gaggctcagctccagc ccacacaactt
MdNPR5_pro_MG CAGCGTCGAGGGCCGTCTCGTCCACACCCACAGGTGCATCCTGGCAGCCTGGAGCCTCTTTTTCTGGAAATTCTTTTGTGGGCCCGAGCCACCGTCGGGT 1400
MdNPR5_pro_MG CAGCGTCGAGGGCCGTCTCGTCCACACCCACAGGTGCATCCTGGCAGCCTGGAGCCTCTTTTTCTGGAAATTCTTTTGTGGGCCCGAGCCACCGTCGGGT 1400
MdNPR5_pro_BG TAGCGTCGAGGGCCGTCTCGTCCACACCCACAGGTGCATCCTGGCAGCCTGGAGCCTCTTTTTCTGGAAATTCTTTTGTGGGCCCGAGCCACCGTCGGGT 1400
MdNPR5_pro_BA TAGCGTCGAGGGCCGTCTCGTCCACACCCACGGGTGCATCCAGGCAGCCTGGAGCCTCTTATTCTGGAAATTCTTTTGTGGGTCAGAGCCACCGTTGGGT 1395
Consensus agcgtcgagggccgtctcgtccacacccac ggtgcatcc ggcagcctggagcctctt ttctggaaattcttttgtggg c gagccaccgt gggt
MdNPR5_pro_MG CTGGATCTCTCGGCGTTGGTGACT 1424
MdNPR5_pro_MG CTGGATCTCTCGGCGTTGGTGACT 1424
MdNPR5_pro_BG CTGGATCTCTCGGCGTTGGTGACT 1424
MdNPR5_pro_BA CTGGATCTATCGGCGTTGGTGACT 1419
Consensus ctggatct tcggcgttggtgact
