# Supplementary material for: Intricate genetic variation networks control the adventitious root growth angle in apple
Source: BMC Genomics. 2020 Dec 1;21:852. doi: 10.1186/s12864-020-07257-8 (PMC7709433; doi:10.1186/s12864-020-07257-8)
Supplement: Supplementary file 12 — Additional file 12: Supplementary File 4 Sequence alignment of MdLBD41 cloned from apple rootstocks ‘BC’ and ‘M9’. (A) CDS. (B) Amino acid. (C) Upstream. [file 12864_2020_7257_MOESM12_ESM.pdf]

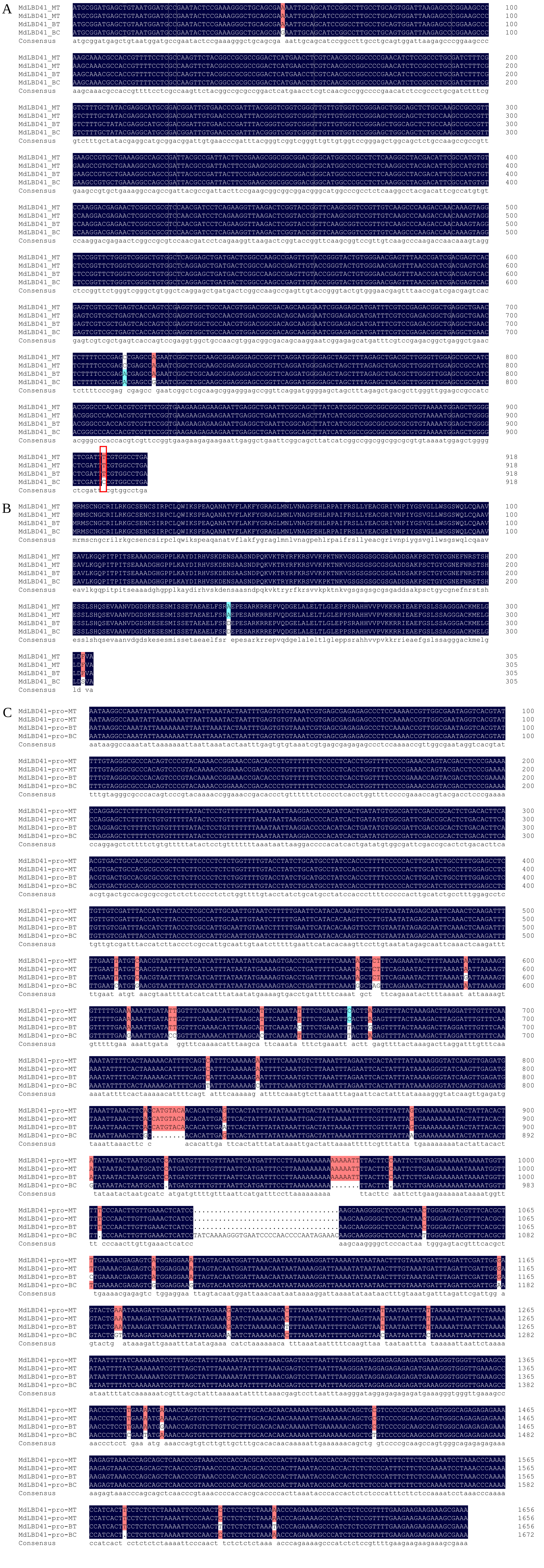
A
MdLBD41_MT
MdLBD41_MT
MdLBD41_BT
MdLBD41_BC
Consensus
B
MdLBD41_MT
MdLBD41_MT
MdLBD41_BT
MdLBD41_BC
Consensus
C
MdLBD41-pro-MT
MdLBD41-pro-MT
MdLBD41-pro-BT
MdLBD41-pro-BC
Consensus
