# Supplementary material for: Intricate genetic variation networks control the adventitious root growth angle in apple
Source: BMC Genomics. 2020 Dec 1;21:852. doi: 10.1186/s12864-020-07257-8 (PMC7709433; doi:10.1186/s12864-020-07257-8)
Supplement: Supplementary file 9 — Additional file 9: Supplementary File 1 Sequence alignment of MdLAZY1 cloned from apple rootstocks ‘Baleng Crab (BC)’ (Malus robusta) and ‘M9’ (M. pumila). (A) Coding sequence (CDS). (B) Amino acid. C: Upstream. [file 12864_2020_7257_MOESM9_ESM.pdf]

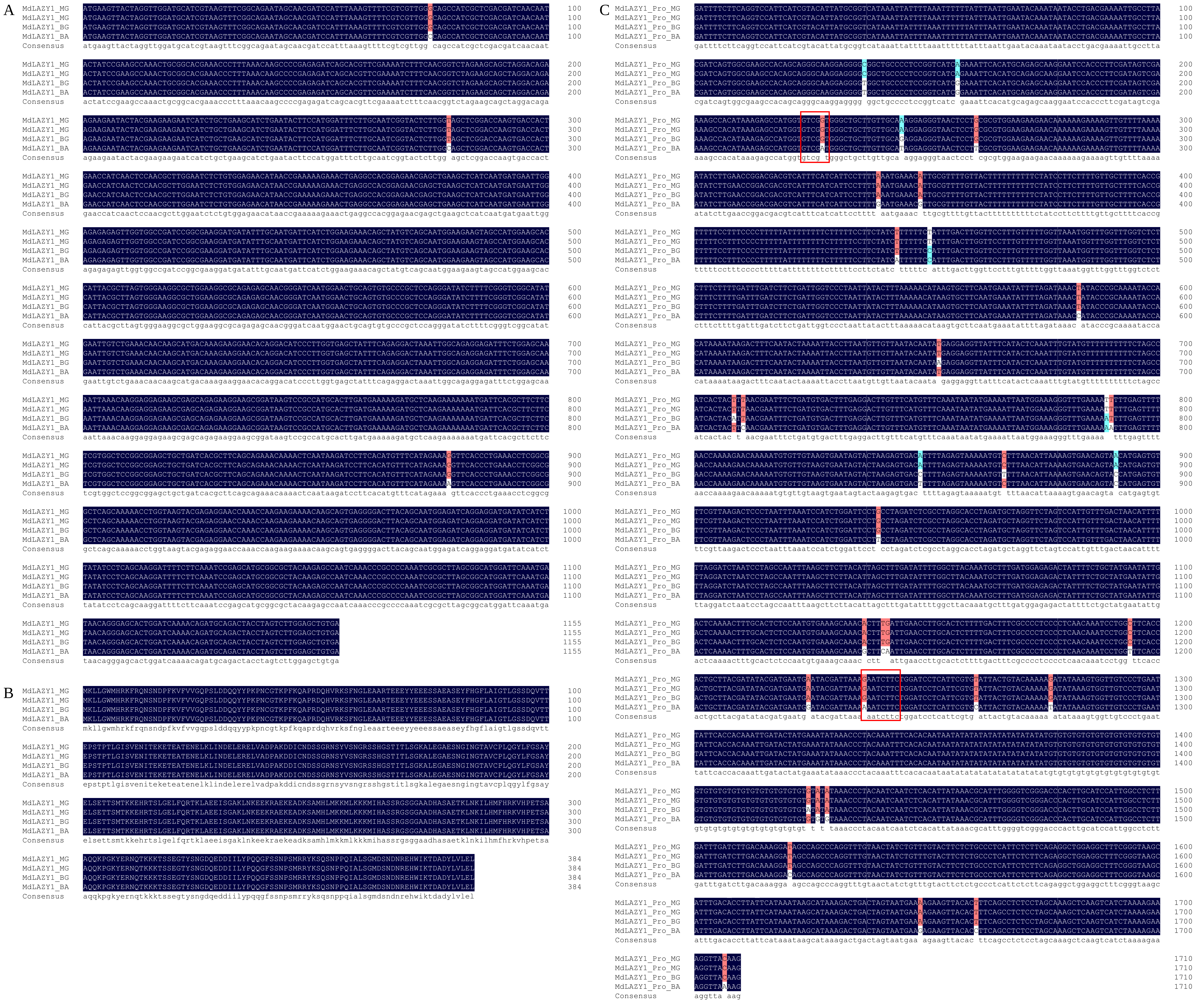
A
MdLAZY1_MG
MdLAZY1_MG
MdLAZY1_BG
MdLAZY1_BA
Consensus
B
C
MdLAZY1_Pro_MG
MdLAZY1_Pro_MG
MdLAZY1_Pro_BG
MdLAZY1_Pro_BA
Consensus
