# Supplementary material for: Intricate genetic variation networks control the adventitious root growth angle in apple
Source: BMC Genomics. 2020 Dec 1;21:852. doi: 10.1186/s12864-020-07257-8 (PMC7709433; doi:10.1186/s12864-020-07257-8)
Supplement: Supplementary file 13 — Additional file 13: Supplementary File 5 Sequence alignment of MdbHLH48 cloned from apple rootstocks ‘BC’ and ‘M9’. (A) CDS. (B) Amino acid. (C) Upstream. [file 12864_2020_7257_MOESM13_ESM.pdf]

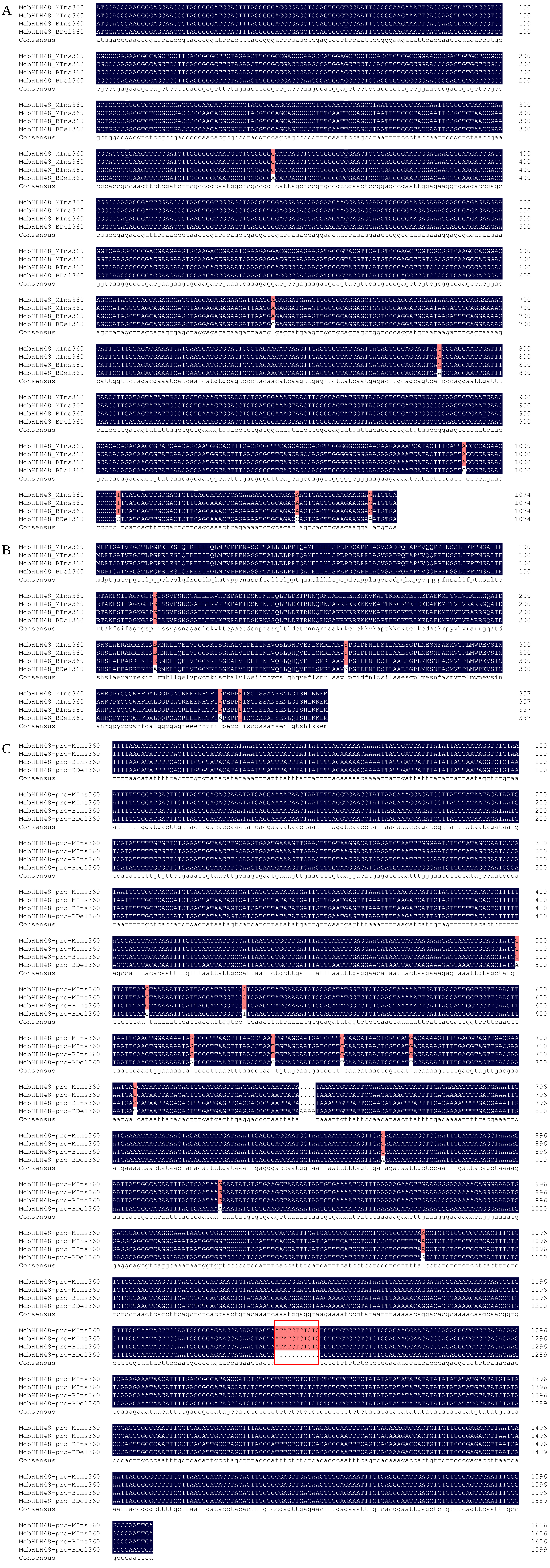
A
B
C
